# Overcoming polyploidy pitfalls: a user guide for effective SNP conversion into KASP markers in wheat

**DOI:** 10.1007/s00122-020-03608-x

**Published:** 2020-06-04

**Authors:** M. Makhoul, C. Rambla, K. P. Voss-Fels, L. T. Hickey, R. J. Snowdon, C. Obermeier

**Affiliations:** 1 Department of Plant Breeding, Justus Liebig University, Giessen, Germany; 2 Queensland Alliance for Agriculture and Food Innovation, The University of Queensland, St Lucia, Australia

## Abstract

**Key message:**

Conversion of SNP chip assays into locus-specific KASP markers requires adapted strategies in polyploid species with high genome homeology. Procedures are exemplified by QTL-associated SNPs in hexaploid wheat.

**Abstract:**

Kompetitive allele-specific PCR (KASP) markers are commonly used in marker-assisted commercial plant breeding due to their cost-effectiveness and throughput for high sample volumes. However, conversion of trait-linked SNP markers from array-based SNP detection technologies into KASP markers is particularly challenging in polyploid crop species, due to the presence of highly similar homeologous and paralogous genome sequences. We evaluated strategies and identified key requirements for successful conversion of Illumina Infinium assays from the wheat 90 K SNP array into robust locus-specific KASP markers. Numerous examples showed that commonly used software for semiautomated KASP primer design frequently fails to achieve locus-specificity of KASP assays in wheat. Instead, alignment of SNP probes with multiple reference genomes and Sanger sequencing of relevant genotypes, followed by visual KASP primer placement, was critical for locus-specificity. To identify KASP assays resulting in false calling of heterozygous individuals, validation of KASP assays using extended reference genotype sets including heterozygous genotypes is strongly advised for polyploid crop species. Applying this strategy, we developed highly reproducible, stable KASP assays that are predictive for root biomass QTL haplotypes from highly homoeologous wheat chromosome regions. Due to their locus-specificity, these assays predicted root biomass considerably better than the original trait-associated markers from the Illumina array.

**Electronic supplementary material:**

The online version of this article (10.1007/s00122-020-03608-x) contains supplementary material, which is available to authorized users.

## Introduction

Today, marker-assisted selection (MAS) for accelerating breeding progress predominantly uses single nucleotide polymorphism (SNP) markers. Commercially available SNP detection technologies for crops include array-based hybridization approaches (predominantly Illumina Infinium arrays and Affymetrix GeneChip/Axiom arrays), PCR-based amplification approaches (for example Kompetitive allele-specific PCR: KASP; LGC Biosearch Technologies, Teddington, UK) or genotyping-by-sequencing approaches like SeqSNP (LGC Biosearch Technologies) or DArT-seq (DArT Pty Ltd, Canberra, Australia) (Obermeier and Friedt 2015; Rasheed et al. 2017; You et al. 2018; Scheben et al. 2018; Hickey et al. 2017). Genome-wide SNP arrays and genotyping-by-sequencing approaches are cost-effective for high marker throughput in combination with moderate sample numbers and are thus mainly used for genetic mapping and genomic selection, which require medium to high throughput with thousands of markers and hundreds to thousands of samples (Mir et al. 2013). However, arrays with fixed marker panels are currently too expensive for targeting of selected subsets of trait-associated markers in breeding programs (Rasheed et al. 2017).

In contrast to these technologies, single-plex KASP markers are more cost-effective to assay low numbers of markers with strong marker-trait associations in thousands of samples, a typical scenario in commercial MAS programs for forward selection approaches (Semagn et al. 2014; Rasheed et al. 2016). KASP, a proprietary genotyping technology of the company LGC Biosearch Technologies, has become widely used by plant breeders for MAS, replacing gel-based molecular marker analyses like simple sequence repeat (SSR) markers during the last decade. Because SNP markers associated with a trait of interest are usually identified using array-based hybridization or genotyping-by-sequencing platforms in genetic mapping studies, they must subsequently be converted into robust breeder-friendly KASP assays to be applicable in applied MAS. The 90 K SNP Wheat iSelect array from Illumina (90 k Wheat Illumina Infinium array) was developed to assess genetic variation in allohexaploid and allotetraploid wheat populations (Wang et al. 2014). Using this array, many studies have been performed to identify marker-trait associations in diverse, homozygous wheat populations (e.g., Gao et al. 2016; Zou et al. 2016; Turuspekov et al. 2017; Voss-Fels et al. 2017). About 30% of the probes from the 90 K Wheat iSelect array show heterozygous clusters for homozygous individuals, due to the presence of homeologous genome sequences hybridizing with individual SNP probes (Wang et al. 2014). SNPs within polyploid genomes that show polymorphism between homeologous sequences from subgenomes (known as inter-homeologue polymorphisms or homeologous SNPs) exhibit heterozygous genotype clusters in single homozygous individuals. If the same SNP probes show homozygous clusters for other individuals and are segregating within a mapping population, they are referred to as ‘hemi-SNPs’ or varietal SNPs and can be used for unambiguous locus-specific genetic mapping in homozygous mapping populations even though the probes themselves are not locus specific (Trick et al. 2009). However, due to the inter-homeologue polymorphism in some individuals, these SNPs cannot distinguish between homozygous and heterozygous individuals and are therefore not useful for commercial breeding (Grewal et al. 2019). Thus, conversion of SNPs from array-based SNP technologies into locus-specific KASP SNP assays able to distinguish heterozygous from homozygous individuals (co-dominant SNPs) is considerably more challenging in polyploid crops compared to diploid crops and requires exhaustive biological and technical validation (Allen et al. 2011, 2013). The aim of this study was to develop strategies and recommendations for successful conversion of Illumina Infinium SNP assays into robust locus-specific KASP assays useful for applied MAS in polyploids. As an example of relevance to wheat breeders, we report on conversion of 15 SNP probes associated with increased root biomass in hexaploid wheat (Voss-Fels et al. 2017). We describe and discuss different aspects of step-by-step KASP marker assay design requirements for polyploids, along with technical and biological validation approaches that are crucial for successful development of locus-specific KASP markers in polyploid wheat and might be extendable to polyploid crop species in general.

## Materials and methods

### Plant material

A panel of 213 genetically diverse hexaploid wheat accessions (described in Voss-Fels et al. 2017) were tested along with approximately 2000 lines from first to fourth generation elite backcross programs aimed at modification of root phenotypes by marker-assisted backcrossing. Total genomic DNA was extracted from 60 to 100 mg of young leaf tissue using the BioSprint 96 DNA Plant kit (Qiagen, Düsseldorf, Germany) according to the manufacturer’s protocol. Artificial heterozygous control samples were obtained by mixing equal amounts of DNA from two divergent homozygous lines, with DNA concentrations measured by Qubit Fluorometric Quantitation (Thermo Fisher Scientific, Waltham, MA, USA).

### KASP assay procedure

All PCR primers were synthesized at Microsynth AG (Balgach, Switzerland) (Supplementary Table S1). The primer mixture comprised 46 μl water, 30 μl common primer (100 μM), and 12 μl of each allele-specific primer (100 μM). KASP assays were performed in 384-well format by using the ViiA7 real-time PCR system (Life Technologies, USA). One KASP reaction volume consisted of a total volume of 5 μl including 2.5 μl DNA (15–25 ng/μl), 0.08 µl primer mixture, and 2.5 µl of KASP master mix with a low ROX level (LGC Biosearch Technologies, UK) or PACE-IR Genotyping Master Mix with a low ROX level (3CR bioscience, Harlow, UK). On each SNP reaction plate, at least one water sample was included as a no-template control. All experiments were repeated at least three times. The thermo-cycling conditions were chosen according to protocols provided by LGC Biosearch Technologies and 3CR bioscience (Supplementary Figure S2). If clear genotyping clusters were not obtained, the plate was subjected to further thermal cycling and re-read until tight genotyping clusters were obtained. The recycle protocol comprised three cycles of denaturation at 94 °C for 20 s and annealing/elongation at 57 °C for 60 s. See also Supplementary File S1 for specific recommendations not addressed in the manufacturer’s manuals which we consider particularly important to design and run locus-specific KASP assays in polyploid species.

### Sequencing of SNP flanking regions from homeologous regions

Locus-specific primers were designed to sequence the target region on each of three homeologous wheat subgenomes, using the program Primer3 v0.4.0 (Untergasser et al. 2012). PCR amplification was performed in 50 µl containing 18.5 µl RNase-free water, 25 µl of GoTaq^®^ Hot Start Colorless Master Mix, (Promega, Madison, WI, USA), 1.5 µl of each primer (10 µM), and 3.5 µl (15–25 ng/μl) genomic DNA. PCR reactions were performed in a T100 Thermal Cycler (Bio-Rad Laboratories, USA) using the following program: primary denaturing at 94 °C for 4 min, followed by 35 cycles of 94 °C for 40 s, 64–60 °C annealing temperature for 40 s and 72 °C for 45 s, with a final extension step at 72 °C for 10 min. Agarose gel electrophoresis was used to separate fragments of PCR products; then, single strong bands of correct size were sequenced from one or both directions using the same forward and reverse primers used for PCR amplification by Microsynth AG using the Sanger sequencing method.

### Sequence data analyses and design of KASP primers

Three different approaches were used. First approach: submission of 101 bp context sequence to LGC Biosearch Technologies for the ‘KASP-by-Design’ service. Second approach: alignment of 101 bp SNP probe sequence to the IWGSC Chinese Spring v1.0 wheat genome reference (IWGSC 2018), The stand-alone BLAST program and a number of online resources, e.g., the BLAST search tool for the IWGSC Chinese spring v0.1 wheat reference sequence at Ensemble Plants (http://plants.ensembl.org/Triticum_aestivum/Tools/Blastwere used for alignment of 90 k SNP flanking 101 bp sequences and 50 bp SNP probe sequences.

The obtained hits were aligned with the multiple sequence alignment tool MUSCLE (https://www.ebi.ac.uk/Tools/msa/muscle/). BLAST hits which high similarity (E-value < 1e–05) were maintained. Sequence similarities were calculated using the Sequence Identity and Similarity tool SIAS (http://imed.med.ucm.es/Tools/sias.html). Based on these multiple alignments, KASP primers were visually placed to unique binding sites. Alternatively, the online tool PolyMarker which uses the IWGSC wheat reference was used (Ramírez-González et al. 2015; PolyMarker 2019). Third approach: alignment of 101 bp SNP probe sequence to multiple resources including the Wheat genome assemblies for the IWGSC Chinese Spring v1, for cv. Paragon (Earlham Inst. v1 scaffolds from Jan 2017, accessed in GrainGenes database 2019) and 10 cultivars from the 10+ Genome Project Wheat Initiative (2019) were used [e.g., at BLAST Search tool for Wheat Collections on GrainGenes (https://wheat.pw.usda.gov/GG3)].

### Statistical data analysis

Correlation analysis between haploblock and root biomass was performed using SPSS Statistics 19.0 software.

### SNP chip data analysis

The Genotyping Module and Polyploid Genotyping Module of the software package GenomeStudio 2.0.4 was downloaded from the Illumina webpage (https://emea.support.illumina.com/array/array_software/genomestudio/downloads.html) and used for SNP chip data clustering and SNP calling from raw data. In addition, we used pre-processed SNP calls processed by the service provider TraitGenetics (Gatersleben, Germany).

## Results

### Evaluation of SNP chip and KASP assay genotyping accuracy by Sanger sequencing

Two haplotype blocks associated with root dry biomass on chromosome 5B of hexaploid wheat were described by Voss-Fels et al. (2017), based on GWAS using the 90 k SNP Illumina Infinium array. Haploblock Hap-5B-RDMa was defined based on SNP calls from 9 SNP chip probes while haploblock Hap-5B-RDMb was defined based on SNP calls from 6 SNP probes. In a set of 215 diverse winter wheat genotypes, 7 different haplotypes were identified for haploblock Hap-5B-RDMa and 9 different haplotypes for haploblock Hap-5B-RDMb (Table 1). Most of the haplotypes for both haploblocks with lower frequencies contained SNP alleles were called by Voss-Fels et al. (2017) as ‘Null’ alleles (10 of 11 below 5% frequency, Table 1) for at least for one probe.

**Table 1:** Comparison of haplotype variants for haploblocks Hap-5B-RDMa and Hap-5B-RDMb detected by SNP chip (left), KASP marker analysis and Sanger sequencing (right) in a panel of 215 wheat genotypes

Genotyping by 90 k SNP chip (Voss-Fels et al. 2017, chromosome 5B, homeologue-specific allele calls by commercial provider)											Genotyping by derived 5B homeologue-specific KASP assays and/or alleles validated by Sanger sequencing										
	Haploblock Hap-5B-RDMa											Haploblock Hap-5B-RDMa									
Haplotype	GENE-2890_482	Excalibur_c25522_755	Kukri_c46570_214	RAC875_c12293_588	RAC875_c18088_2222	BobWhite_c43_86	RAC875_c18088_950	RAC875_c24226_1356	Excalibur_c60554_394	Frequency	Haplotype	GENE-2890_482	Excalibur_c25522_755	Kukri_c46570_214	RAC875_c12293_588	RAC875_c18088_2222	BobWhite_c43_86 (HapA6-2)	RAC875_c18088_950	RAC875_c24226_1356	Excalibur_c60554_394 (HapB9-2)	Frequency
h1	G	C	A	G	G	G	C	A	G	86.5	h1	G	C	A	G	G	G	C	A	G	91.6
**h2**	A	T	G	A	A	A	T	C	T	8.4	**h2**	A	T	G	A	A	A	T	C	T	8.4
h3	G	C	A	G	G	G	–	A	G	1.9	h1	G	C	A	G	G	G	C	A	G	
h4	G	C	A	G	G	G	C	–	G	1.4	h1	G	C	A	G	G	G	C	A	G	
h5	G	C	A	G	G	G	C	A	–	0.9	h1	G	C	A	G	G	G	C	A	G	
h6	G	C	A	G	G	–	C	A	G	0.5	h1	G	C	A	G	G	G	C	A	G	
h7	–	C	A	G	G	G	–	–	–	0.5	h1	G	C	A	G	G	G	C	A	G	

	Haploblock Hap-5B-RDMb								Haploblock Hap-5B-RDMb						
Haplotype	BS00022231_51	BS00022477_51	BS00029852_51	BS00110293_51	IACX6288	Tdurum_contig48959_1172	Frequency	Haplotype	BS00022231_51	BS00022477_51 (HapB2-2)	BS00029852_51 (HapB3-2)	BS00110293_51	IACX6288 (HapB5-2)	Tdurum_contig48959_1172	Frequency
h1	G	A	G	A	T	G	74.4	h1	G	A	G	A	T	G	79.6
h2	A	G	T	G	C	G	13.5	h2	A	G	T	G	C	G	14.4
**h3**	G	G	T	G	C	T	5.1	**h3**	G	G	T	G	C	T	5.6
h4	G	A	–	A	T	G	4.2	h1	G	A	G	A	T	G	
h5	A	G	T	G	–	G	0.9	h2	A	G	T	G	C	G	
h6	G	G	T	G	–	T	0.5	h3	G	G	T	G	C	T	
h7	G	A	G	A	–	G	0.5	h1	G	A	G	A	T	G	
h8	G	A	G	G	C	T	0.5	h8	G	A	G	G	C	T	0.5
h9	G	–	–	A	T	G	0.5	h1	G	A	G	A	T	G	

KASP assay names are given in brackets used to detect null allele (on the right). The combination of haplotype h2 in haploblock Hap-5B-RDMa and haplotype h3 in haploblock Hap-5B-RDMb shown in bold letters is associated with high root biomass

The low frequency of some haplotypes harboring ‘Null’ alleles suggested that the failed calls in SNP chip genotyping are either due to hybridization or SNP calling artifacts and not due to genuine ‘Null’ alleles resulting from presence/absence variation for the 50 bp region complementary to the SNP probe. To test this hypothesis in a case study, KASP assays were designed for two affected SNP probes of Hap-5B-RDMa and for all 3 affected SNP probes of Hap-5B-RDMb (Table 1). These assays were applied to 19 genotypes including different haplotype combinations for Hap-5B-RDMa and Hap-5B-RDMb. In contrast to the SNP chip genotyping results, none of the 5 tested KASP assays failed to call one of the two major alleles, neither did they ever called a ‘Null’ allele (Table 1 on the right). However, this might also be due to amplification of the allele-specific primer from a matching complementary nucleotide following the ‘Null’ allele position. To confirm the correct allele composition of these genotypes, the SNP flanking sequences were analyzed by Sanger sequencing for all 23 genotypes harboring genuine ‘Null’ alleles that covered the full sequence complementary to 8 probes. Sanger sequencing revealed that all ‘Null’ alleles were incorrectly called by SNP array genotyping and that the KASP assays always called the correct alleles (Supplementary Table S3).

**Fig. 1:**
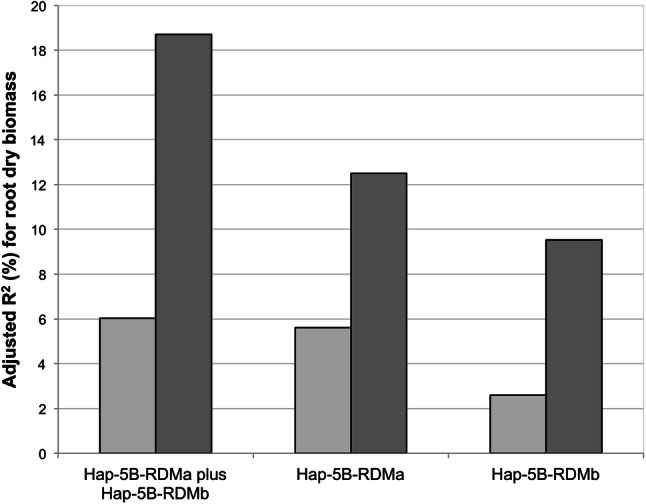
Correlation coefficient (adjusted R^2^) of haploblocks Hap-5B-RDMa and Hap-5B-RDMb variants for root dry biomass in 215 wheat genotypes using SNP calls from 90 k SNP array data (light gray) and the corresponding variants called by KASP markers confirmed with Sanger sequencing (corrected haplotypes, dark gray)

Applying the corrected genotype data from the KASP marker analysis, the number of haplotype variants was reduced from seven to two observed haplotypes within haploblock Hap-5B-RDMa (Hap-5B-RDMa-h1 and -h2) and from nine to four haplotypes within haploblock Hap-5B-RDMb (Hap-5B-RDMb-h1, -h2, -h3 and -h8) (Table 1). Only one of 11 putative low-frequency haplotypes (< 5%) was confirmed by KASP genotyping and Sanger sequencing (h8 for haploblock Hap-5B-RDMb, frequency 0.5%). Recalculation of the haploblock-trait association for root dry biomass using the corrected KASP marker genotyping data increased the correlation coefficient (adjusted R^2^) from 5.6% to 12.5% for haploblock Hap-5B-RDMa and from 2.6 to 9.5% for haploblock Hap-5B-RDMb (Fig. 1). Also, linkage disequilibrium analysis based on the correlation of newly developed KASP marker pairs for the panel of Voss-Fels et al. (2017) revealed no indication that the organization of the markers associated with the trait in two haploblocks on chromosome 5B is not correct.

### Identification of trait-associated nucleotide alleles and calling errors from SNP chip hybridization data

**Fig. 2:**
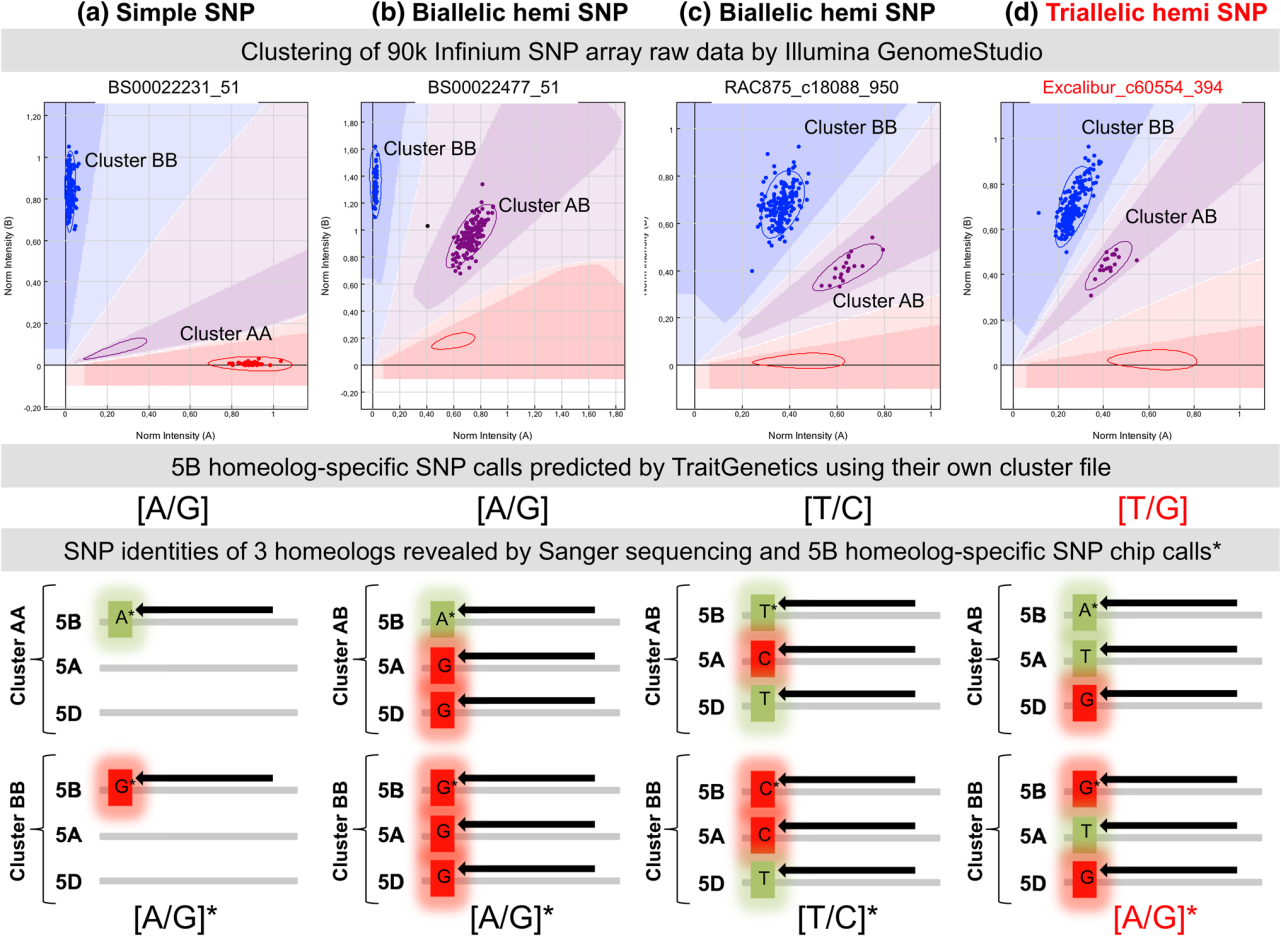
Comparison of SNP chip clustering plots from software GenomeStudio, SNP prediction/calling and probe alignment specificities (for two example genotypes) for simple SNP and hemi-SNP probes on chromosome 5 of homozygous hexaploid wheat accessions. **a** Simple SNP where the probe binds specifically to the 5B homeolog. **b** Biallelic non-homeologous hemi-SNP probes, where all probes bind to 3 homeologs producing two clusters (1 heterozygous and 1 homozygous). **c** Biallelic homeologous hemi-SNP probes where the probes bind to all 3 homeologs producing two heterozygous clusters. **d** Triallelic hemi-SNP probe with two heterozygous clusters, where prediction of a polymorphism on 5B homeologue resulted in an incorrect T/G call when relying on customer probe data, whereas Sanger sequencing revealed a 5B homeologue-specific A/G polymorphism

Conversion of SNP chip markers used in GWAS and biparental QTL mapping into breeder-friendly marker systems requires determination of the nucleotide allele identity associated with the increase/decrease in the trait of interest, sourced either from published studies or from SNP chip databases. However, allele identification is not always simple due to the use of different formats applied for reporting the SNP and allele identities. For example, in the CropSNP database (http://snpdb.appliedbioinformatics.com.au), genotype data for Illumina wheat Infinium array SNP calls are reported either as AA/AB/BB allele patterns (representing cluster positions) or as predicted nucleotide allele patterns (e.g., AA, AT, TT) (Scheben et al. 2019). Some commercial service providers also report the allele patterns in IUPAC one letter code (e.g., A, W, T). In addition, if using a commercial service provider, the raw data might not be provided and processed SNP calls are reported to the customer based on a cluster file developed or optimized by the company (e.g., providing SNP calls cleaned from hemi-SNP calling patterns, including processing artifacts). Also, most publications on GWAS or biparental QTL mapping do not contain enough additional information to directly infer the SNP probe composition and SNP nucleotide allele identity associated with an increase/decrease in a target trait to derive other marker types. One reason is that strand-specific identity for a SNP allele cannot unambiguously be reported for hybridization probes, as correct orientation of contigs within genome assemblies might be adjusted over time and is dependent on specific reference genome assemblies. To ensure consistency in reporting SNP allele calls from SNP chip assays, Illumina uses their own TOP/BOT strand nomenclature and method (Illumina technical note 2006; Nelson et al. 2012; Zhao et al. 2018). Furthermore, the 50 bp SNP probe sequences for Illumina Infinium arrays are rarely directly published. Instead, a minimum of 101 bp sequences flanking the SNP polymorphism (with 50 bp on the left and 50 bp on the right of the SNP, e.g., Wang et al. 2014, Supplementary Table S5) is usually published, and this sequence is used by Illumina to design the final 50 bp probe sequences. To identify the exact 50 bp probe sequences used on the SNP chip (e.g., for BLAST analyses to a reference genome), the TOP/BOT designations for the submitted customer strand and for the Illumina design strand are provided in the manifest file from Illumina. This information can be accessed in the GenomeStudio software by importing the raw hybridization data (idat color files), sample and chip information (sample sheet, manifest file; ‘Customer Strand’ and ‘ILMN Strand’ columns in the SNP table). We applied these rules for identification of called alleles for the 15 root biomass-associated SNPs detected by the SNP chip array and validated the SNP chip calls by Sanger sequencing. Examples are given in Supplementary Table S2 for identification of SNP probe sequence composition. In diploid organisms, biallelic loci are expected to exhibit three cluster positions for a simple SNP (AA, AB, and BB; Fig. 2a). Based on the customer-submitted biallelic SNP identities, these clusters can be assigned to homozygous and heterozygous nucleotide calls following the Illumina TOP/BOT nomenclature by using information from the manifest file (see rules described in The Triticeae Toolbox T3 for details, https://triticeaetoolbox.org/wheat/). A summary of the data and the derived SNP alleles associated with high root dry biomass on the respective DNA strands are presented in Table 2. However, especially for polyploids like hexaploid bread wheat, observed clusters and predicted nucleotide allele designations do not always represent the homozygote and/or heterozygote states and correct nucleotide allele identities. Instead, they only represent the position of the clusters against the x and y axis, defined by the GenomeStudio software applying the manifest file provided by Illumina (plus any customized adaptations by the customer or a genotyping service provider). SNP array variant calling for 15 SNP probes associated with root biomass in wheat in Table 1 was performed by the commercial service provider TraitGenetics, applying a custom cluster file adapted using a large panel of world-wide wheat accessions. Non-polymorphic hemi-alleles were removed by TraitGenetics from the SNP call information and chromosome-specific nucleotide alleles from these example SNPs on chromosome 5B are predicted from the clustering patterns of each SNPs (Supplementary Figure S1, Fig. 2b–d). For 99% of the data points, these SNP calls were identical with SNP calls from the raw Illumina data files and the standard manifest file in GenomeStudio (after removing the 5A and 5D chromosome signal calls from the hemi-SNPs and translation into the IUPAC single-letter code). However, these SNP nucleotide call predictions were not always correct for one or both data sets. In 1 out of 15 cases for the 15 SNP assays we investigated, Sanger sequencing revealed that a different nucleotide allele combination should have been called and a different nucleotide than reported is associated with increase in wheat root biomass. This is shown for probe Excalibur_c60554_394 in Fig. 2d. Here, a T/G polymorphism was called and based on this data a T allele was predicted to be associated with high root biomass for haplotype 5B-RDMa-h2 (based on the predicted customer SNP variation submitted by the customer to Illumina). In contrast, Sanger sequencing of the 5B homeolog revealed a A/G polymorphism and based on this data an ‘A’ allele was found to be associated with high root biomass (Table 2). Sanger sequencing of the 5A and 5D homeologs and comparison with the wheat reference genome revealed that the SNP probe Excalibur_c60554_394 is specific for all three homeologous copies of the reference genome, but the 5A and 5D homeologs were found by Sanger sequencing to be monomorphic between all tested homozygous wheat accessions. In contrast, the 5B homeologue was found to be polymorph between accessions segregating with an A/G polymorphism. Thus, the polymorphism is 5B homeologue-specific and is detected by a typical hemi-SNP cluster pattern. However, as ‘A’ and ‘T’ are both measured by the same green color signal (Cy3), while ‘G’ and ‘C’ are both measured by the same red color signal (Cy5) in the dual-color Illumina detection system, the nucleotide segregation on chromosome 5B for a hemi-SNP is measured by a dosage-dependent clustering with two heterozygous clusters. Hence, when relying solely on the submitted customer SNP information embedded in the manifest file, this SNP is incorrectly predicted as a T/G polymorphism, instead of the correct ‘A/G’ polymorphism, due to the underlying ‘triallelic’ hemi-SNP (Fig. 2d). One cluster in Fig. 2d represents the ‘AATTGG’ (5B, 5A, 5D) allele composition (4 green signal, 2 red signal equivalents) for haplotype h2 (associated with high root biomass) with the ‘AA’ alleles coming from homeologue 5B. The other cluster in Fig. 2d represents the ‘GGTTGG’ allele composition (2 green, 4 red signal equivalents) for the low root biomass haplotypes, with two of the four ‘GG’ alleles derived from homeologue 5B. Prediction of allele composition for clusters observed in SNP chip data based on the homeologue-specificity of the SNP evaluated by genetic mapping and a reference genome might call incorrect nucleotide alleles for hemi-SNPs due to the dual-color nature of the detection system. Note that the cluster positions for some biallelic hemi-SNPs with correct SNP call predictions cannot be distinguished from triallelic hemi-SNPs with false SNP call predictions (Fig. 2c, d). Accordingly, when using the predicted T/G polymorphism for probe Excalibur_c60554_394 to design allele-specific KASP primers, we failed four times to achieve the expected clusters until we redesigned primers based on SNP flanking sequences of all 3 homeologs in 15 different genotypes produced by Sanger sequencing.

**Table 2:** SNP calls produced from raw SNP chip data using the software GenomeStudio and by a commercial SNP chip genotyping service provider (SP) and comparison with Sanger sequencing of regions flanking SNPs in up to six homeologous and paralogous copies of probe targets (5B1, 5B2, 5A1, 5A2, 5D1, 5D2); §Nucleotide alleles for the haplotype combination Hap-5B-RDMa-h2 and Hap-5B-RDMb-h3 associated with high root biomass are shown in bold letters

	Data from GenomeStudio		Processed data received from SP	Sanger sequencing using homeolog-specific primers and alignment to multiple reference assemblies/genomes						Allele associated with high root biomass	Conclusion from data comparison
SNP probes sorted by haploblocks	SNP call	Cluster plots in Figure	Reported alleles	5B1, major haplotypes§	5B2	5A1	5A2	5D1	5D2		SNP probe type, SNP homeolog specificity
Haploblock Hap-5B-RDMa				Hap-5B-RDMa-							
GENE-2890_482	[AA/AB]	S1	A/G	h1: G; **h2: A**	–	A	–	A	–	A	hemi-SNP, 5B1
Excalibur_c25522_755	[AB/BB]	S1	T/C	h1: C; **h2: T**	C	T	C	C	C	T	hemi-SNP, 5B1
Kukri_c46570_214	[AB/BB]	S1	A/G	h1: A; **h2: G**	G	G	–	G	–	G	hemi-SNP, 5B1
RAC875_c12293_588	[AB/BB]	S1	A/G	h1: G; **h2: A**	–	G	–	A		A	hemi-SNP, 5B1
RAC875_c18088_2222	[AB/BB]	S1	A/G	h1: G; **h2: A**	–	G	–	G	–	A	hemi-SNP, 5B1
BobWhite_c43_86	[AA/BB]	S1	A/G	h1: G; **h2: A**	–	A	–	A	–	A	simple SNP, 5B1
RAC875_c18088_950	[AB/BB]	S1, 2	T/C	h1; C; **h2: T**	–	C	–	T	–	T	hemi-SNP, 5B1
RAC875_c24226_1356	[AB/BB]	S1	A/C	h1: A; **h2: C**	C	C	–	C	–	C	hemi-SNP, 5B1
Excalibur_c60554_394	[AB/BB]	S1, 2	T/G	h1: G; **h2: A**	–	T	–	G	–	A	hemi-SNP, 5B1
**Haploblock Hap-5B-RDMb**				Hap-5B-RDMb-							
BS00022231_51	[AA/BB]	S1, 2	A/G	h2: A;h1, **h3**, h8**:G**;	–	–	–	–	–	A	simple SNP, 5B1
BS00022477_51	[AB/BB]	S1, 2	A/G	h1, h8: A; h2, **h3: G**	–	G	–	G	–	G	hemi-SNP, 5B1
BS00029852_51	[AA/BB]	S1	T/G	h1, h8: G; h2, **h3: T**	–	T	–	–	–	T	simple SNP, 5B1
BS00110293_51	[AA/AB/BB]	S1	A/G	h1: A; h2, **h3,** h8**: G**	–	–	–	–	–	G	unclear, 5B1
IACX6288	[AB/BB]	S1	T/C	h1: T; h2, **h3,** h8**: C**	–	C	–	C	–	C	hemi-SNP, 5B1
Tdurum_contig48959_1172	[AA/BB]	S1	T/G	h1, h2: G; **h3**, h8**: T**	–	–	–	–	–	T	simple SNP, 5B1

– indicates no copy detected (similarity below 80%). S1 indicates Supplementary Figure S1

### Conversion rate into KASP assays using SNP probe flanking regions for primer design

Three successive approaches exhibiting increasing data analysis complexity were applied to convert SNPs detected by SNP arrays into valid predictive KASP assays (Table 3). In the first, simplest approach, the SNP flanking sequences (101 bp) submitted to Illumina for probe design (Supplementary Table S2) were used to design primers from KASP assays without any further consideration of the wheat genome composition. This is the approach applied by the commercial KASP assay design service (‘KASP-by-Design’) of LGC Biosearch Technologies. However, the standard genotyping service provided by LGC does not accept common and allele-specific primer sequences as input in their submission form. Instead, a minimum of 101 bp sequencing flanking the SNP are required as input (e.g., the sequence used to design the SNP probe by Illumina). When providing LGC the 101 bp flanking regions of the 15 SNPs associated with root biomass, without masking any region for primer design (Supplementary Table S2), 11 out of 15 assays produced clearly separated KASP clusters, but only 3 of 15 assays from haploblock Hap-5B-RDMb produced KASP clusters showing genotype SNP calling patterns consistent with the SNP chip data, using a reference genotype set of 213 lines (Table 3). This suggests a putative lack of locus-specificity of the designed KASP assays.

**Table 3:** Conversion rates for SNPs spanning both haploblocks Hap-5B-RDMa and Hap-5B-RDMb from SNP chip arrays into validated KASP marker assays

Number of successful KASP assays resulting in cluster positions expected for 213 reference genotypes when primer design was based on	SNPs in both haploblocks	SNPs in haploblock Hap-5B-RDMa	SNPs in haploblock Hap-5B-RDMb
1st approach: 101 bp SNP flanking sequences	3 of 15	0 of 9	3 of 6
2nd approach: 101 bp aligned to 3 subgenomes of single reference genome (IWGSC RefSeq v1.0)	8 of 15	2 of 9	6 of 6
3rd approach: 101 bp aligned to multiple wheat genomic resources plus Sanger sequencing of flanking regions for about 40 selected reference genotypes	11 of 15	5 of 9	6 of 6

**Fig. 3:**
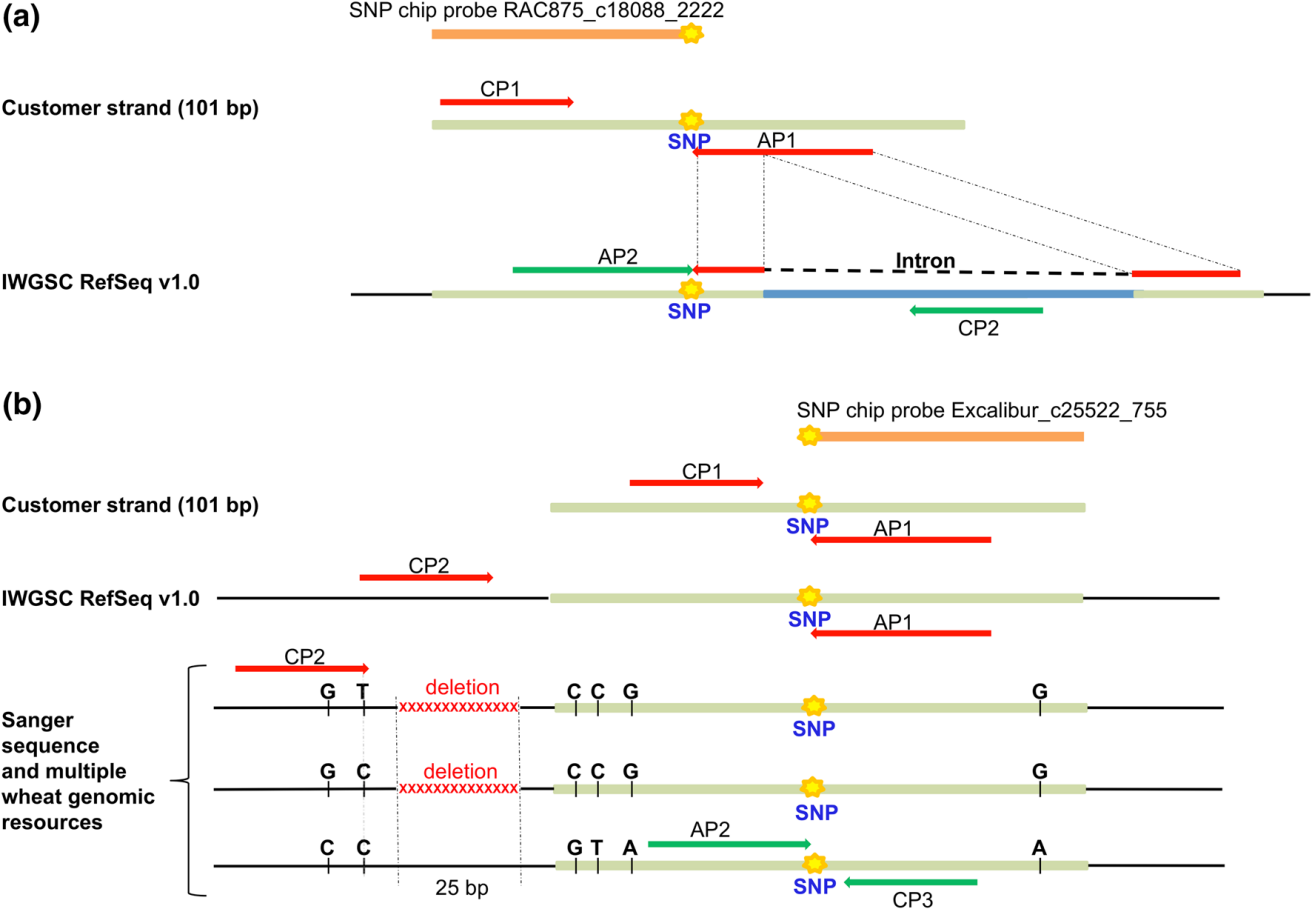
Examples for successful and unsuccessful strategies to convert two SNPs detected by the SNP probes RAC875_c18088_2222 (**a**) and Excalibur_c25522_755 (**b**) into KASP markers. *AP* allele-specific primers, *CP* common primer. In red color: KASP primer pairs which failed; in green color: KASP primer pairs which worked (color figure online)

One reason for this low conversion rate is that the probe flanking sequences used to design probes for the 90 K SNP chip are derived from RNA-Seq data and not from genomic data. Ignoring this might result in the design of primer sequences aligning across exon–intron boundaries within the wheat genome, consequently leading to a subsequent failure of the KASP assays (no signals). An example is shown in Supplementary File S2 (example 2). BLASTn analysis of the 101 bp SNP flanking sequences against the wheat reference genome IWGSC RefSeq v1.0 cv. Chinese Spring confirmed that 5 out of 9 SNP-containing sequences in haploblock Hap-5B-RDMa and 0 out of 6 SNP-containing sequences in Hap-5B-RDMb were interrupted by introns (Supplementary Table S4, example in Fig. 3a). This explained the assay conversion failure for 3 of these 5 SNP probes in haploblock Hap-5B-RDMb, in the remaining 2 the primer binding sites were placed just by chance outside of intron sequences. Also from the 12 SNP probes not KASP primers flanked by introns only 3 were successfully converted into KASP assays. Comparison of these sequence alignment data and SNP chip calling data from GenomeStudio (Supplementary Figure S1) with the obtained KASP cluster patterns revealed that only the 3 SNP probes showing high similarity to only one homeologue on chromosome 5B (Supplementary Table S4) and a simple SNP segregation pattern in SNP chip data produced clearly separated expected clusters in KASP assays with 213 genotypes. These 3 successful KASP assays were derived from the 101 bp flanking sequence for probes BS00022231_51, BS00110293_51, and Tdurum_contig48959_1172 (haploblock Hap-5B-RDMb).

### Conversion rate into KASP assays using SNP probe flanking regions and comparative alignment to wheat reference genomes/assemblies for primer design

In a second approach (Table 3), the KASP assays were redesigned based on the obtained comparative alignment with the IWGSC RefSeq Chinese Spring v1.0 reference genome, avoiding intron spanning primer binding sites and locus-unspecific common primer binding sites by visual primer placement. This resulted in an increase in successful KASP assays so that the 3 remaining SNPs located within the haploblock Hap-5B-RDMb could be converted into validated KASP assays.

**Fig. 4:**
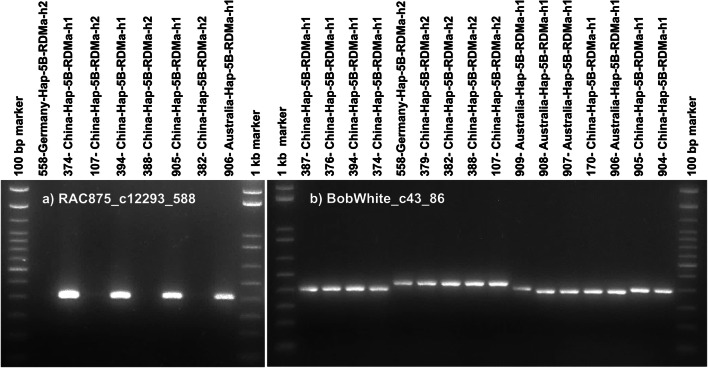
PCR products obtained using primers which were designed flanking two SNPs (**a**, **b**) in haploblock Hap-5B-RDMa, based on the wheat IWGSC v1.0 Chinese Spring reference genome

In contrast, only 2 out of 9 SNPs (Kukri_c46570_214, RAC875_c18088_2222) in haploblock Hap-5B-RDMa could be successfully converted into KASP assays producing the expected clusters when applying the second approach (Supplementary Table S4). From the 7 remaining KASP assays designed for haploblock Hap-5B-RDMa using this approach, 5 produced clearly separated clusters. However, although they were able as expected to call all 213 homozygous reference samples, they failed to call heterozygous samples correctly. To explore why the seven KASP assays designed using the 2nd approach for haploblock Hap-5B-RDMa failed to call genotypes correctly, 5B subgenome-specific primer pairs for Sanger sequencing of the SNP flanking regions (300–650 bp) were designed using the Chinese Spring reference genome and software package Primer3. These primers were used for PCR amplification from 40 lines harboring haplotypes Hap-5B-RDMa-h1 and -h2 (Table 4). For Hap-5B-RDMb, PCR amplification products of the expected sizes were always obtained for genotypes exhibiting haplotypes Hap-5B-RDMb-h1, -h2, -h3 and -h8. In contrast, for haploblock Hap-5B-RDMa the expected PCR products were always obtained for genotypes exhibiting haplotype Hap-5B-RDMa-h1, but in the majority of cases (7 out of 9 SNPs) not for genotypes exhibiting haplotype Hap-5B-RDMa-h2. Within the region flanking SNP BobWhite_c43_86, a length polymorphism between haplotypes Hap-5B-RDMa-h1 and -h2 was detected by gel electrophoresis and Sanger sequencing (Figs. 4, 8).

**Table 4:** Summary of results for PCR amplification for Sanger sequencing of SNP flanking regions using subgenome-specific primer pairs designed based on two different genome assemblies (IWGSC Chinese Spring v0.1 and Paragon)

	Subgenome-specific primer pairs for Sanger sequencing designed based on the IWGSC v0.1 reference								
	haploblock Hap-5B-RDMa								
Haplotype	GENE-2890_482	Excalibur_c25522_755	Kukri_c46570_214	RAC875_c12293_588	RAC875_c18088_2222	BobWhite_c43_86	RAC875_c18088_950	RAC875_c24226_1356	Excalibur_c60554_394
h1	+	+	+	+	+	+	+	+	+
h2	–	–	–	–	+	+	–	–	–

	Subgenome-specific primer pairs for Sanger sequencing designed based on Paragon scaffolds								
	haploblock Hap-5B-RDMa								
Haplotype	GENE-2890_482	Excalibur_c25522_755	Kukri_c46570_214	RAC875_c12293_588	RAC875_c18088_2222	BobWhite_c43_86	RAC875_c18088_950	RAC875_c24226_1356	Excalibur_c60554_394
h1	*n*	*n*	*n*	*n*	*n*	*n*	*n*	*n*	*n*
h2	+	+	+	+	*n*	n	+	+	+

	Haploblock Hap-5B-RDMb					
Haplotype	BS00022231_51	BS00022477_51	BS00029852_51	BS00110293_51	IACX6288	Tdurum_contig48959_1172
h1	+	+	+	+	+	+
h2	+	+	+	+	+	+
h3	+	+	+	+	+	+
h8	+	+	+	+	+	+

	Haploblock Hap-5B-RDMb					
Haplotype	BS00022231_51	BS00022477_51	BS00029852_51	BS00110293_51	IACX6288	Tdurum_contig48959_1172
h1	*n*	*n*	*n*	*n*	*n*	*n*
h2	*n*	*n*	*n*	*n*	*n*	*n*
h3	*n*	*n*	*n*	*n*	*n*	*n*
h8	*n*	*n*	*n*	*n*	*n*	*n*

(+) indicates PCR amplification product of the expected size was obtained using primers flanking this SNP probe location for genotypes harboring this haplotype, (−) indicates no PCR amplification product was obtained for genotypes harboring this haplotype, (*n*) indicates no genotypes harboring this haplotype were tested

To explore why Sanger sequencing failed for genotypes exhibiting haplotype Hap-5B-RDMa-h2 seven subgenome-specific primer pairs were redesigned flanking the SNP using scaffold sequences from accession Paragon (Earlham Inst. v1) which also harbors haplotype 5B-RDMa-h2. PCR amplification products were obtained for all genotypes exhibiting haplotype Hap-5B-RDMa-h2 (Table 4). Based on the Sanger sequencing results KASP primers were designed along with multiple genomic resources. This led to a further increase in the number of successfully converted KASP assays from 2 to 5 out of 9 in haploblock Hap-5B-RDMa (Table 3, Supplementary Table S4, example 1 in Supplementary File S2). For the remaining 4 SNPs in haploblock Hap-5B-RDMa, the high subgenome similarity and polymorphism between reference genome/assemblies around the SNP position in haploblock Hap-5B-RDMa prevented conversion into locus-specific KASP assays.

### Identification of reasons for incorrect cluster calling and strategies for improvement of clarity and locus-specificity

**Fig. 5:**
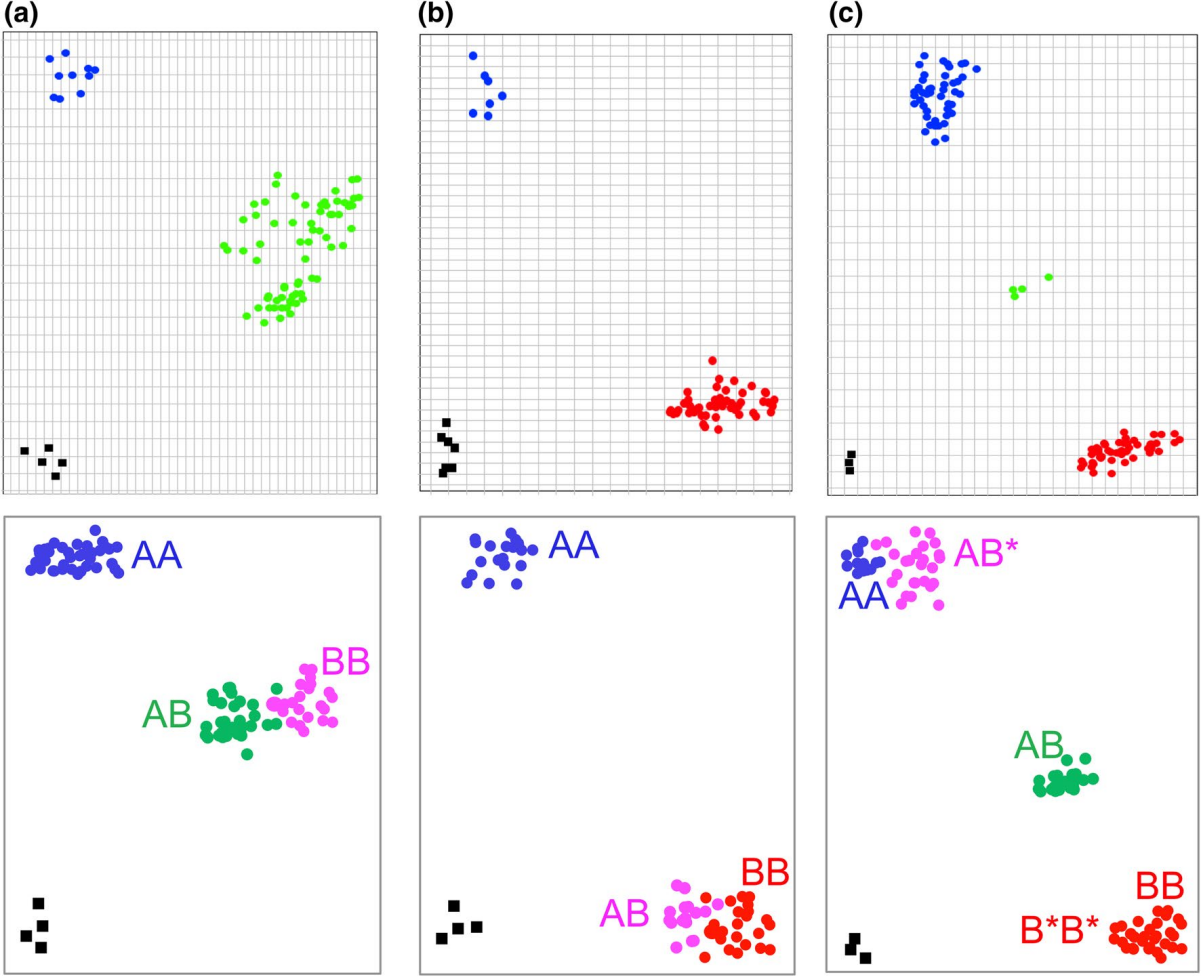
Schematic representation of different scenarios in which unexpected KASP clusters were obtained; **a** all homozygous genotypes carrying allele B are clustering with a heterozygous cluster AB due to the common primer binding to more than one locus (hemi-SNP). **b** All heterozygous genotypes AB are clustering with a homozygous cluster BB due to preferential amplification of allele B over allele A (for example, mismatch at 3′ end of common primer binding site or PCR product size differences resulting from short deletions). **c** Some heterozygous genotypes AB* are clustering with a homozygous cluster AA due to a deletion at 5′ end of common primer binding site in genotypes carrying allele B*

When testing initially designed KASP primer combinations with a set of 213 reference genotypes with known allele composition, 5 out of 9 assays for haploblock Hap-5B-RDMa and 3 out of 6 for haploblock Hap-5B-RDMb produced unexpected KASP clusters. Sanger sequencing of a set of 80 affected genotypes revealed different reasons that some genotypes were assigned to the wrong clusters (Fig. 5). A case which is often reported in the literature is a typical hemi-SNP shown in Fig. 5a, where all homozygous genotypes for one allele are clustering in a ‘heterozygous’ cluster due to the common primer binding to more than one subgenome. The other two cases of wrong clustering identified by Sanger sequencing were due to a mismatch of the 3′ end of the common primer site for one allele, a deletion at the 5′ end of the common primer or a size difference between the two allelic PCR products (Fig. 5b, c). All these cases result in preferential binding, amplification of one allele relative to another, and false calling of heterozygous genotypes as homozygous or vice versa.

**Fig. 6:**
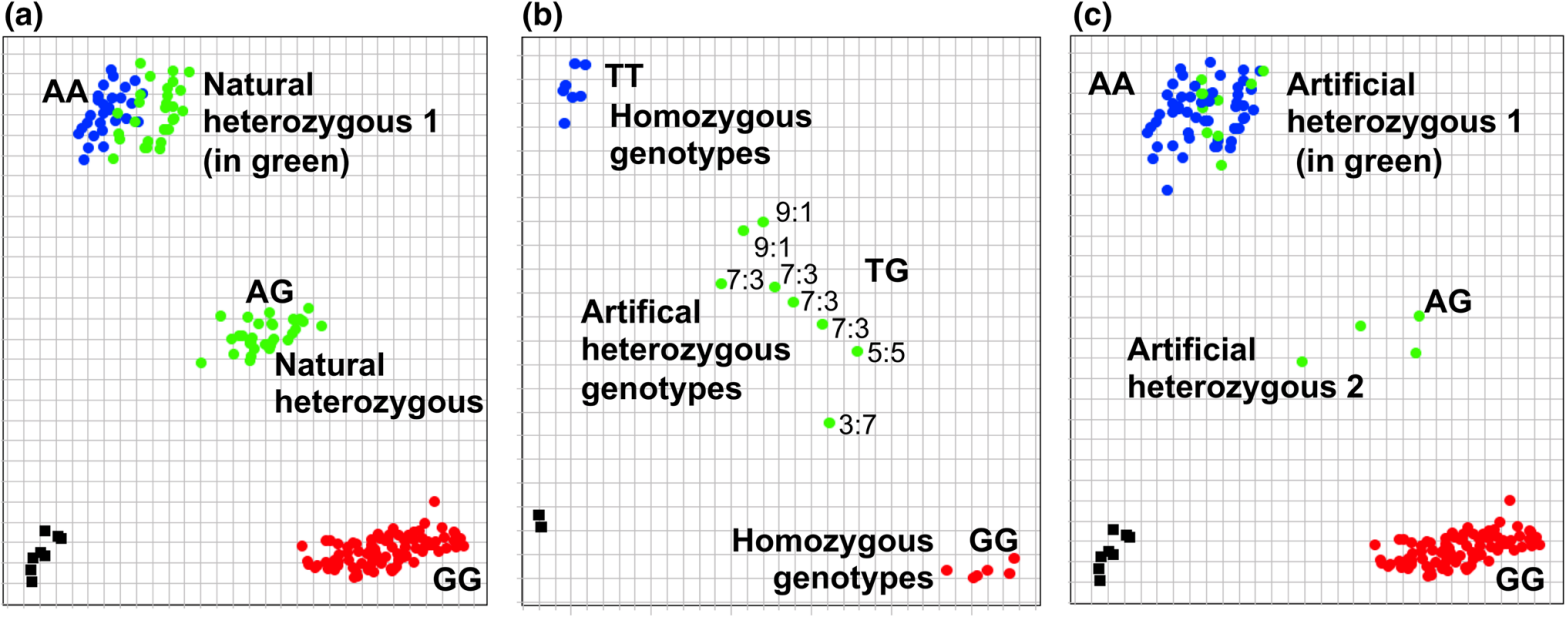
Example validation for KASP assay HapA6-1 using heterozygous F1 individuals (**a**) and artificial heterozygous DNA samples (**b**, **c**) for identification of assays resulting for some genotypes in false cluster calling (**c**, here crosses from parent P1 and P2). **b** Artificial heterozygous DNA samples over a wide range of ratios (9:1–1:9 of TT/GG allele carrying homozygous DNA samples) are correctly identified as heterozygous cluster (green) and called as TG alleles (color figure online)

False SNP call assignments for some heterozygous genotypes is particularly problematic if broadly applicable locus-specific KASP assays need to be developed for MAS. Figure 6a shows as an example for KASP assay HapA6-1, initially derived from SNP probe BobWhite_c43_86 in Haploblock Hap-5B-RDMa. Some heterozygous F1 genotypes from a cross of parents P1 × P2 are clustering falsely (AA, in green) together with the homozygous parent P1 (AA, in blue). Figure 6b shows that, if no heterozygous F1 reference genotypes are available, artificial mixtures of DNA from two divergent homozygous genotypes (P1 and P2) will cluster reliably in the expected heterozygote pattern over a wide range of concentration ratios between 1:9 and 9:1. Figure 6c shows that using artificial heterozygous DNA samples from parents P1 and P2, instead of natural heterozygote plants, can also allow identification of false clustering.

### Optimization of KASP markers associated with root biomass for MAS

**Fig. 7:**
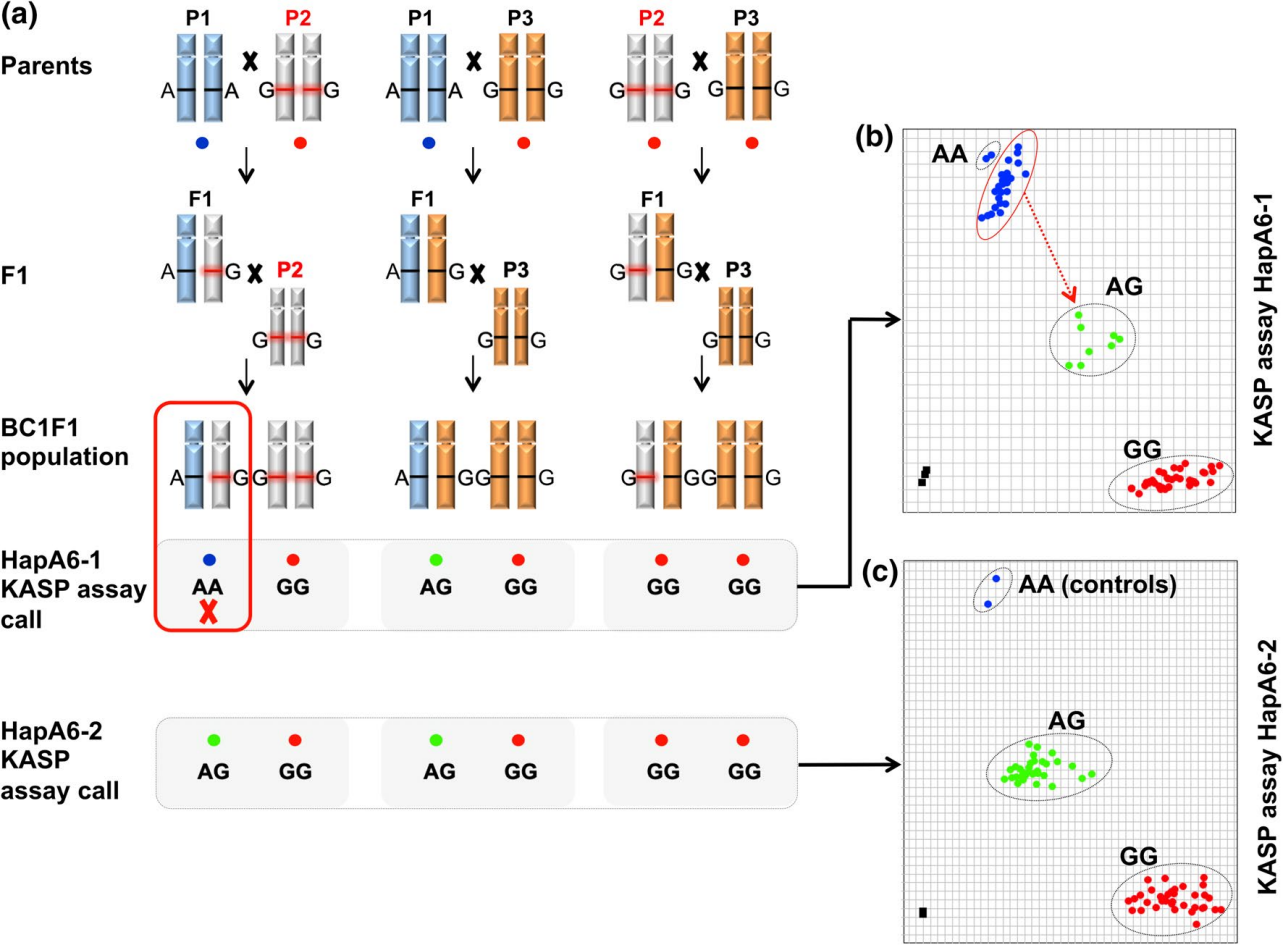
Optimization of KASP assays for a backcrossing program. **a** Breeding scheme and allele calling using two different KASP assays. **b** Initially designed KASP assay HapA6-1 showing wrong clustering of some heterozygous genotypes (BC1F1 from parent 1 and parent 2), **c** redesigned KASP assays HapA6-2 adapted to the breeding program with correct calling of heterozygous BC1F1 offspring. Parent P2 represents an Australian elite cultivar with alleles associated to low root biomass

**Fig. 8:**
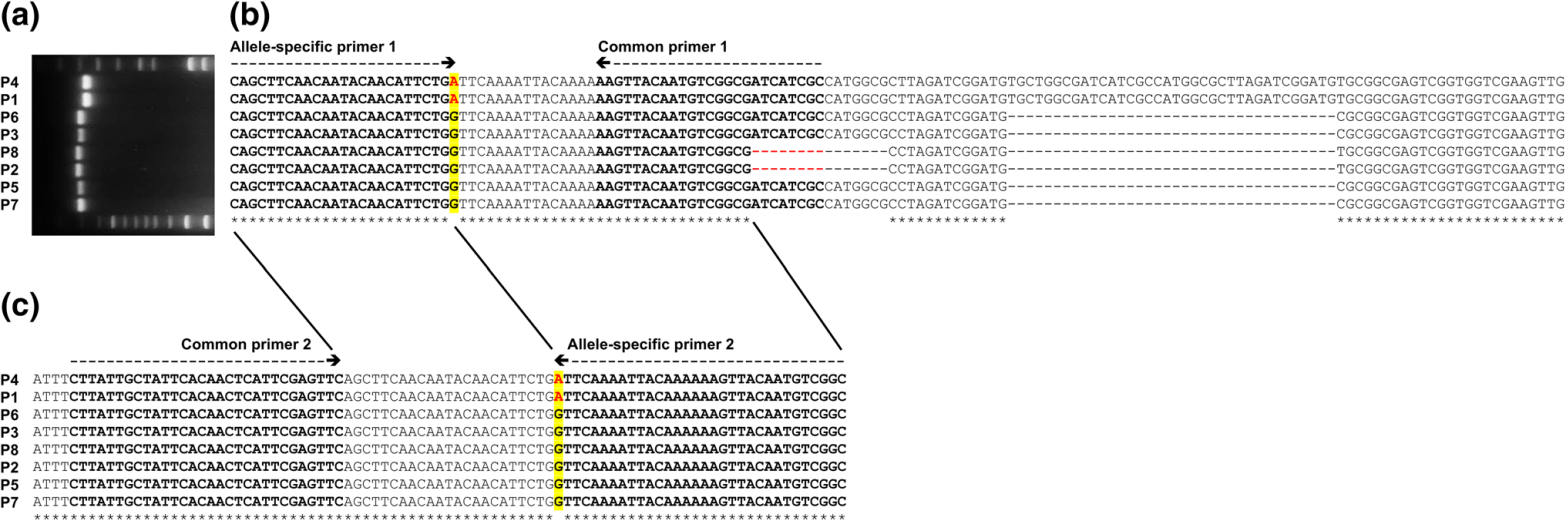
**a** PCR products, and **b**, **c** Sanger sequence alignments obtained from primers, based on IWGSC v1.0 Chinese Spring wheat reference genome, designed to flank a SNP detected by probe BobWhite_c43_86 in haploblock Hap-5B-RDMa, amplified from 8 genotypes used as parents in the backcrossing program. Parents P8 and P2 show a deletion of 53 bp compared to Parents P4 and P1. The other four parents show a 36 bp deletion. Primer binding sites of allele-specific and common primers for KASP assay HapA6-1 (**b**) resulting in false genotype calls and optimized KASP assay HapA6-2 (**c**) resulting in correct genotype calls are shown

In total, 11 KASP assays were derived from two haploblocks and validated in 213 reference genotypes from major wheat growing regions of the world (Supplementary Figure S2). To test these assays for use in a breeding program, extensive validation in breeding material was applied to evaluate the robustness and limitations of the assays. The KASP assays were evaluated in a backcrossing program and applied for marker-assisted selection for increased root dry biomass. Of the 11 KASP assays, 3 were predicted to be sufficient to distinguish the haplotype combination Hap-5B-RDMa-h2 and Hap-5B-RMDb-h3 associated with high root biomass from other haplotype combinations associated with low root biomass (HapA6-1 for haploblock Hap-5B-RDMa; HapB3-2 and HapB6-1for haploblock Hap-5B-RDMb). In the backcrossing program, two parents with high root biomass originating from China, 2 parents from the Netherlands with low root biomass and 4 other parents representing adapted cultivars from Australia were used. In total, approximately 400 offspring were tested with the 3 selected KASP assays. Two of the assays produced stable and robust data (HapB3-2, HapB6-1). However, one KASP assay, HapA6-1, produced unexpected clustering results in the F1 and BC1F1 generations, revealing a homozygous cluster including a high number of genotypes with high root biomass in F1 and BC1F1 (Fig. 7b). Sanger sequencing of 8 parents was performed for the primer target site. Two genotypes used as parents in the breeding program revealed an 8 bp deletion at the common primer binding site (parental cultivars P2 and P8 in Fig. 8). Optimization and redesign of the KASP assay into the assay HapA6-2 produced the expected clusters (Fig. 7c) and was successfully adapted to the backcrossing program for high root biomass selection.

## Discussion

Compared to diploid species, the highly similar structure of homeologous subgenomes in relatively recent polyploid crop species like hexaploid bread wheat is expected to limit the development of broadly applicable locus-specific SNP assays. However, although many reports exist on the validation of SNP markers in different polyploid crops, very different criteria have been used for judging the quality of markers and these are mostly insufficiently defined. Also, the comprehensiveness of technical validation and terms used to describe the quality of markers can vary substantially. Platten et al. (2019) suggested to judge the quality of a molecular marker based on a number of core and supporting metrics, including three main categories: technical, biological and breeding metrics. Here, we follow this classification with some modifications. Technical metrics, including call rate and clarity, were determined as the percentage of samples giving a visible result (i.e., not ‘missing’), while clarity was the percentage of samples giving a clearly scorable and correctly scored result with known genotypes (as opposed to unclear or ambiguous results). The original SNP chip data reported in Voss-Fels et al. (2017) and the derived KASP assays for the same SNPs in the present study both gave call rates of 100%. LGC Biosearch Technologies reports typical call rates for KASP assays of over 95% across all species (https://www.biosearchtech.com/support/faqs/kasp-genotyping-assays/what-are-the-typical-call-rates-for-kasp-genotyping). However, call rates alone are of limited use to judge marker quality. This can be seen in our study, where although the call rates were 100% for both SNP technologies, the allele calls using these two different technologies were conflicting for 1.1% of data points, indicating that technical accuracy of SNP calling varies between the two methods. Thus, we suggest adding the criterion ‘calling accuracy,’ or ‘accuracy based on independent assessment,’ to the set of technical metrics of Platten et al. (2019) for judging marker quality. We validated the accuracy of the SNP calling based on the array and based on the derived KASP assays, by comparison of SNP calls obtained by Sanger sequencing. Sanger sequencing confirmed that the SNP calls from all KASP assays were technically correctly called. In contrast, all ‘Null’ alleles called from 90 K Wheat SNP chip hybridization data were technically incorrectly called and should have instead been called as ‘missing’ data. However, it should be noted that from the Illumina Infinium 60 k Brassica SNP chip, a fraction of the ‘missing’ data has been found to allow reliable calling of ‘Null’ alleles or presence/absence variation by population-based quality filtering in polyploid *Brassicas napus* (Gabur et al. 2018), indicating that independent validation of haplotypes harboring ‘Null’ alleles should be performed. If no independent validations are performed haplotypes including ‘Null’ alleles and exhibiting low frequencies should be considered to be genotyping artifacts (e.g., in Qian et al. 2017; Alahmad et al. 2019). Removing them from analysis would putatively result in more correct haplotype definitions and consequently in biologically more accurate haplotype-trait associations. Even though only 1.1% of data points were conflicting between the SNP chip and KASP genotyping in the present study, the construction of haplotypes including these incorrect data points in the SNP chip data analysis resulted in about 6% frequency for incorrectly defined haplotypes, having a strong effect on the correct marker-trait association with an strong underestimation and difference of > 200% (up to 12% difference in R^2^). In addition, exclusion of incorrectly predicted ‘Null’ alleles will lower the number of required KASP markers necessary to distinguish genotypes carrying favorable combinations of haplotype variants for application in MAS.

We found that the percentage of KASP markers giving a clearly scorable and accurate result with known genotypes can vary substantially when using different approaches to design KASP primers. The simplest approach to design KASP markers from SNP chip data using only 101 bp SNP flanking sequences revealed a low conversion rate of 20%, and therefore is not recommended for polyploid species. This approach is also applied when using the commercial LGC KASP-by-Design service. Furthermore, the second approach, using a single genome reference for alignment of SNP flanking regions before primer design, is not recommended as it increased the success rate for valid KASP marker design for one haploblock, Hap-5B-RDMa, to only 22%. The reason for this low success rate was identified by Sanger sequencing of a large number of genotypes with different haplotype composition to be a high sequence similarity of haploblock Hap-5B-RDMa on chromosome 5B to homeologous and paralogous genomic regions on chromosomes 5A and 5D and polymorphism between genotypes in the targeted genome region. This indicates that the wheat reference genome IWGSC v1.0 is sufficient to cover the most common genome composition for some regions, e.g., for haploblock Hap-5B-RDMb in the wheat gene pool, but is not sufficient to cover the diversity in other regions, e.g., haploblock Hap-5B-RDMa of the wheat gene pool. Thus, it is highly recommended for polyploid species to apply our third tested approach for KASP marker conversion from SNP chip data using a reference genome plus multiple genome assemblies (and if necessary Sanger sequencing from selected genotypes of the population) for alignment of SNP flanking regions and placement of KASP primers to unique binding sites.

Following multiple genome alignments and identification of unique sequence regions flanking a SNP of interest, KASP primer design might be performed with a primer design program, like Primer3, using the target region as input. However, Primer3 and other primer design programs often failed to design appropriate primers at the intended unique genome positions, because they use a short single input sequence even when masking common regions. Such programs are generally optimized to base primer selection primarily on restrictive thermodynamic calculations, predictions of self-complementarity and other parameters rather than positional restrictions. In contrast, many primer pairs which are excluded by these primer design programs worked perfectly under laboratory conditions in KASP assays after visual design and exclusive selection for unique primer binding sites (e.g., allele-specific primers in Fig. 8c harboring ‘A’ nucleotide homopolymers rejected by Primer3). The semiautomated and commonly used KASP online design program PolyMarker (PolyMarker 2019; Ramírez-González et al. 2015), which is specifically adapted for polyploid species using a single whole genome alignment approach, did not perform well in locus-specific primer design for the 15 SNP probes we tested. PolyMarker suggested for 15 SNP probes used as input 3 KASP assays which were considered specific to the target locus on chromosome 5B. In contrast, 9 KASP assays were reported to be either semi-specific or nonspecific. For another 3 SNP probes PolyMarker reported that no primer design was possible. One reason for poor performance is that PolyMarker cannot source primers from multiple alignment input files using more than one reference genome as input at a time. Another reason might be that PolyMarker uses the primer design program Primer3 with standard restrictive parameters and adjustment of the KASP assay primer requirements for Primer3 is not possible using the online version of PolyMarker. In contrast, non-automated alignment of all similar subgenomic regions of the IWGC reference genome using MUSCLE, visual inspection and moving of common primer binding sites by a few base pairs allowed us to design locus-specific assays for 14 out of the same 15 input sequences. Thus, design of KASP primers by visual inspection from multiple alignments requires a more laborious step-by-step processing, but is also still more efficient for a low number of assays than using primer design programs applying restrictive non-adapted parameters. Automated tools for KASP marker assay which use multiple reference genome alignments to predict locus-specific primers might become available in the near future, as more and more genome assemblies are published (e.g., see software development described in Alsamman et al. 2019).

The technical marker quality metrics ‘clarity’ includes the percentage of samples giving a clearly scorable result, and the percentage of samples giving correctly scored results compared to reference genotypes with defined allele composition (accuracy). However, quality validation for SNP and other markers especially with respect to accuracy is very rarely reported in the literature. A common problem we identified when converting SNP chip into KASP assays in hexaploid wheat was the production of unexpected clusters with the reference genotypes. We also realized that within 9 out of 15 cases where KASP markers produced unexpected clusters, the original SNP probes from the array also created segregation clusters typical for hemi-SNPs indicated by homo- and heterozygous cluster positions in allelic color discrimination plots for homozygous genotypes. Hemi-SNP segregation patterns in chip genotyping do not limit the use of this data for locus-specific genetic mapping in homozygous mapping populations. In this case, the raw data can be processed by calling the inter-varietal polymorphism (e.g., A/R segregation) and/or removing the ambiguous SNP calls known from genetic mapping studies and reference genome alignment (e.g., by translation of A/R segregation into 5B homeolog-specific A/G segregation calls). However, applying SNP chip genotyping data from SNP probes creating hemi-SNP type segregation patterns cannot be applied for heterozygous genotypes, as differences in varietal variation cannot be distinguished from homeologous variation. This prevents the use of this SNP type for MAS. For this reason, researchers usually try to avoid using hemi-SNPs for KASP marker conversion for breeding. Instead, single-copy regions of the wheat genome can be addressed by using only markers with unique genome positions, which avoids difficulties in distinguishing between heterozygous and homozygous genotypes (e.g., Allen et al. 2013; Grewal et al. 2019). However, this strategy biases genotyping toward genome regions without strong homoeology, and may cause a lack of tightly linked markers for a trait of interest if causal genes lie in highly homoeologous regions (as in the example we describe here). Thus, for polyploid species, it is highly recommended before attempting to design locus-specific KASP marker primers, to inspect the raw idat-file color data in GenomeStudio, and for SNPs showing hemi-SNP segregation patterns to apply sequencing of homeo-alleles to improve success rate for KASP assay conversion.

The present study is focusing on KASP marker conversion form Illumina iSelect 90 K array probes. Another SNP array technology platform commonly used by researchers and breeders is the Affymetrix Axiom platform (e.g., Axiom Wheat Breeder’s 35 K and Wheat HD 817 K genotyping arrays). SNP detection by both technologies is based on the same principle, the hybridization by complementary base pairing and signal intensity capture of fluorescent probes. Hybridization specificity on Illumina arrays is based on 50 bp probes, whereas on Affymetrix arrays it is based on one or two 20 bp probes and thus the Affymetrix Axiom technology is more tolerant to any additional variants present in the probe sequence compared to Illumina Infinium technology (You et al. 2018). Thus, the approaches and recommendations given here for conversion of KASP assays from Illumina data are also relevant to design locus-specific KASP markers from Axiom arrays. Supplementary File S1 contains a summary of our experiences and recommendations which we consider particularly important to design and run locus-specific KASP assays in polyploid species from SNP arrays.

A common problem in hexaploid wheat in KASP marker conversion we identified was the frequent false calling of heterozygous genotypes (5 out of 15 KASP assays). Sanger sequencing of primer target sites for these assays suggested that preferential amplification of one allele over the other in KASP assays is highly sensitive to incomplete binding of one of the allele-specific primers, due to polymorphisms (including deletions) between genotypes in primer binding sites or due to size difference between the two allelic PCR products (e.g., Neelam et al. 2013). Thus, validation for KASP assay accuracy by including known (or artificial) heterozygote samples is very crucial. Artificial heterozygous samples have been used in performance evaluation and quality control in clinical molecular genetics, where they showed a similar ability to detect heterozygote alleles as in our study (Jarvis et al. 2005; Coggins et al. 2017). Surprisingly, we have found that most recent studies reviewing and reporting ‘validation’ of KASP markers in crops did not use heterozygous genotypes for validation of accuracy as a proof for marker validation (e.g., Rasheed et al. 2016; Tan et al. 2017a, b; Singh et al. 2019). Based on our results, we suggest that inclusion of natural or artificial heterozygous DNA samples should be a key requirement in all KASP marker validation studies in crops.

## Conclusions

This study reports a set of basic rules for conversion of SNP chip markers into breeder-friendly, robust, locus-specific KASP markers for MAS in hexaploid wheat. Technically and biologically robust KASP assays for polyploid species urgently require primer design based on alignments with multiple reference genome assemblies, Sanger sequencing of parental lines of breeding populations and inclusion of reference genotypes from diverse origins. Use of natural or artificial heterozygous reference DNA samples as controls on each KASP assay plate is recommended to obtain well-separated clusters and identify assays causing false clustering for some heterozygous genotypes due to variation in primer binding sites.

## Electronic supplementary material

Below is the link to the electronic supplementary material.

Supplementary_Tables_S1_to_S4.xlxs (XLSX 40 kb)

KASP assay recommendations for polyploid species (PDF 121 kb)

Examples for stepwise conversion of SNPs from the 90k Illumina wheat SNP array into locus-specific KASP assays (PDF 385 kb)

90k SNP chip discrimination plots for SNP probes located in haploblocks Hap-5B-RDMa and Hap-5B-RDMb for 213 genotypes of a wheat diversity panel (PDF 3639 kb)

KASP discrimination plots for KASP assays located in haploblocks Hap-5B-RDMa and Hap-5B-RDMb for 213 genotypes of a wheat diversity panel (PDF 193 kb)
