# Supplementary material for: Overcoming polyploidy pitfalls: a user guide for effective SNP conversion into KASP markers in wheat
Source: Theor Appl Genet. 2020 Jun 4;133(8):2413–30. doi: 10.1007/s00122-020-03608-x (PMC7360542; doi:10.1007/s00122-020-03608-x)
Supplement: Supplementary file 2 — KASP assay recommendations for polyploid species (PDF 121 kb) [file 122_2020_3608_MOESM2_ESM.pdf]

Theoretical and Applied Genetics

Overcoming polyploidy pitfalls: A user guide for effective SNP conversion into KASP markers in wheat

Makhoul M[1], Rambla C[2], Voss-Fels KP[2], Hickey LT[2], Snowdon RJ[1], Obermeier C[1]

[1]Department of Plant Breeding, Justus Liebig University, Giessen, Germany

[2]Queensland Alliance for Agriculture and Food Innovation, The University of Queensland, St Lucia, Australia

KASP assay recommendations for polyploid species

KASP assays were designed and run according to manufacturer's manuals and recommendations (KBioscience 2011; LGC Limited 2013, 2014a, 2014b, 2015; 3CR Bioscience Ltd. 2018). Below we list some of our specific technical experiences and recommendations not addressed in the manufacturer's manuals which we consider particularly important to design and run locus-specific KASP assays in polyploid species based on the experiences with hexaploid wheat and tetraploid oilseed rape.

1. The position of the 3' end of the allele specific primers is fixed when converting assays from SNP arrays into KASP assays. However, the allele specific primers can either be positioned to be complementary to the upper or to the lower strand. In hexaploid wheat the high similarity between homoeologues genomes mostly results in alignment of the allele specific primers to sequence regions in all three sub-genomes and sometimes also to paralogous regions. Therefore, the specificity of the KASP assay depends mainly on selecting common primer binding sites with one or more nucleotide differences at 3`end of the primer to ensure locus-specificity (anchoring of primers, see LGC Limited, 2015). Thus, the position of the 3' end of the primers is an absolute restrictive criterion. Primer design programs with standard parameters are intending to find the most suitable primer in a longer sequence region of a single DNA sequence usually using a combination of criteria giving different penalties to a number of criteria, instead of one absolute criterion and thus should be avoided or run with adapted relaxed parameters and by anchoring of the primer binding sites for design of locus-specific KASP assays for polyploid species.

2. The melting temperature of common and allele specific primers can be adjusted by using allele specific and common primers of different length. For hexaploid wheat we recommend to design KASP primers with a melting temperature (T*m*) within a range of 63°C to 67°C calculated by the software Primer3.

3. We used two different KASP master mixes, from LGC Biosearch Technologies (KASP master mix with a low ROX level) and from 3CR bioscience (PACE-IR Genotyping Master Mix Low ROX), according to the manufacturer's recommendations and did not find differences in sensitivity and performance. For both master mixes we recommend to use instead of the standard program of LGC Biosearch Technologies (LGC Limited, 2011) their 65-57°C touchdown protocol (LGC Limited, 2014a), but with a minimum of 30 cycles as also recommended by the 3CR Bioscience (3CR Bioscience Ltd., 2018).

4. LGC Biosearch Technologies recommends an amount of DNA from 5 to 50 ng per reaction (LGC Limited, 2013) and 3CR Bioscience an amount of 1 to 10 ng per reaction (3CR Bioscience Ltd., 2018) depending on the genome size. For hexaploid wheat we recommend an amount of genomic DNA from 40 to 80 ng per reaction quantified using Qubit. Signals will still be obtained for wheat with DNA amounts down to 0.25 ng. However, samples with a low genomic DNA concentration require additional PCR cycles sometimes resulting in scattered clusters. Also, too high amounts of genomic DNA negatively affect the correct clustering in the KASP allelic discrimination plot and thus it is important to keep the DNA concentration below 130 ng per reaction for hexaploid wheat.

5. Rasheed *et al.* (2016) reported that a slightly higher concentration of the allele-specific primer with the HEX cassette improved the KASP results for wheat. We did not find that an increase of the concentration of any of the allele-specific primers changed or improved the location of clusters on a discrimination plot. We also did not found changes in sensitivity and clustering when the unique tail sequence of the allele specific primers that corresponds to the HEX cassette was replaced with the one which corresponds to the FAM cassette, and vice versa.

6. LGC Biosearch Technologies and 3CR Bioscience both recommends for every KASP plate to run two No-Template-Controls (NTC) together with the samples. In addition to this, they advices to include positive controls i.e. DNA samples of known genotype especially when working with a SNP that has low allele frequency. In addition, LGC Biosearch Technologies recommends running a minimum of 22 samples on each KASP plate to ensure correct clustering (LGC Limited, 2013). We strongly recommend for every KASP plate a minimum of 22 samples plus a minimum of 2 known homozygous reference genotypes of allele A and allele B each plus 2 known heterozygous AB reference genotypes to ensure correct clustering of all tested genotypes in the allelic discrimination plot. KASP plates should never be run without heterozygous genotypes to ensure correct clustering. We observed, that it is quite common that homozygous reference genotypes are called as heterozygous in wheat. This may indicate a lack of locus-specificity of the designed KASP assay and underlines the importance of using heterozygous reference samples on every KASP plate. If no heterozygous reference genotypes are available artificial heterozygous samples can be produced by mixing equal amounts of genomic DNA of different homozygous genotypes. Even if a ratio of 9:1 of one genotype over the other is produced this will result in clusters in the

discrimination plot which will be clearly positioned between the two homozygous clusters. If the allelic discrimination plot shows that heterozygous samples are called as homozygous for allele B, this might indicate that a polymorphism exists in the allele specific primer binding site for allele A or a difference in amplicon lengths leading to preferential amplification of allele B relative to allele A in heterozygous samples. If for homozygous reference samples the allelic discrimination plot shows that homozygous samples which carry the allele A have comparable fluorescent values with the NTC samples, while homozygous samples which carry the allele B have elevated fluorescent values, this might indicate that a polymorphism at the primer binding site exists on the sequence which carries allele A.

7. LGC Biosearch Technologies recommends to anchor common primers within 70 bp of the target SNP (LGC Limited, 2015) which corresponds to a maximum amplicon length of about 150 bp including HEX or FAM tails. In our hands KASP assay amplicon lengths from 142 to 187 bp including HEX or FAM tails worked well and gave the expected SNP calls for our reference genotype set. However, when the amplicon length is above 160 bp including HEX or FAM tails then the number of cycles in the should be increased up to 38.

8. LGC Research Technologies and 3CR Bioscience recommend to recycle the plate for a few cycles if after the standard KASP cycling program no tight clusters are formed (LGC Limited, 2013, 2014b, 3CR Bioscience Ltd., 2018). However, too many additional cycles might result in merging clusters, unassigned or wrongly assigned reference samples and interpretations of recycled plates should be done carefully. The number of PCR recycling steps required to obtain distinct clusters varies from one assay to another depending on many factors mainly related to quantity and quality of DNA and to the structure of the target region (%GC content, amplicon length).

References

3CR Bioscience Ltd. (2018) PACE-IR™ Genotyping Master Mix User Guide v1.2. https://3crbio.com/wp-content/uploads/2020/01/PACE-IR-User-Guide-v1.2.pdf. Accessed 2 January 2020

KBioscience (2011) KASP version 4.0 SNP Genotyping Manual v1.001. https://www.cerealsdb.uk.net/cerealgenomics/CerealsDB/PDFs/KASP_SNP_Genotyping_Manual.pdf. Accessed 2 January 2020

LGC Limited (2013) KASP genotyping chemistry User guide and manual. https://biosearch-cdn.azureedge.net/assetsv6/KASP-genotyping-chemistry-User-guide.pdf. Accessed 2 January 2020

LGC Limited (2014a) KASP thermal cycling conditions. https://biosearch-cdn.azureedge.net/assetsv6/KASP-thermal-cycling-conditions-all-protocols.pdf. Accessed 2 January 2020

LGC Limited (2014b) Guide to running KASP genotyping reactions on the ABI StepOne and StepOnePlus instruments (ABI ViiA7). https://biosearch-cdn.azureedge.net/assetsv6/running-KASP-on-ABI-StepOne-and-StepOnePlus.pdf. Accessed 2 January 2020

LGC Limited (2015) KASP assay design – Anchoring. https://biosearchassets.blob.core.windows.net/assetsv6/guide_kasp-assay-design-anchoring.pdf. Accessed 17 September 2019

Rasheed A, Wen W, Gao F.M, Zhai S, Jin H, Liu JD, Guo Qi, Zhang Y, Dreisigacker S, X Xianchun, H Zhonghu (2016) Development and validation of KASP assays for functional genes underpinning key economic traits in wheat. Theor Appl Genet 129:1843-1860
