# Supplementary material for: Overcoming polyploidy pitfalls: a user guide for effective SNP conversion into KASP markers in wheat
Source: Theor Appl Genet. 2020 Jun 4;133(8):2413–30. doi: 10.1007/s00122-020-03608-x (PMC7360542; doi:10.1007/s00122-020-03608-x)
Supplement: Supplementary file 3 — Examples for stepwise conversion of SNPs from the 90k Illumina wheat SNP array into locus-specific KASP assays (PDF 385 kb) [file 122_2020_3608_MOESM3_ESM.pdf]

Overcoming polyploidy pitfalls: A user guide for effective SNP conversion into KASP markers in wheat

Makhoul M[1], Rambla C[2], Voss-Fels KP[2], Hickey LT[2], Snowdon RJ[1], Obermeier C[1]

[1]Department of Plant Breeding, Justus Liebig University, Giessen, Germany

[2]Queensland Alliance for Agriculture and Food Innovation, The University of Queensland, St Lucia, Australia

Examples for stepwise conversion of SNPs from the 90k Illumina wheat SNP array into locus-specific KASP assays

This document describes two examples for stepwise conversion of SNP assays into locus-specific KASP assay using three different approaches. Typically encountered problems and solutions are described in detail.

Example 1: SNP probe Excalibur_c25522_755 from haploblock Hap-5B-RDMa

1) First approach

101 bp flanking the SNP detected by probe Excalibur_c25522_755 (Figure 1) were submitted according to the requirements to the company LGC Biosearch Technologies for their "KASP by Design" service. This service designs KASP primers without any further consideration of the wheat genome composition. The primer information is usually not released to the customer by the company. However, we received the primer information shown in Figure 2 for optimization of the KASP assay.

Excalibur_c25522_755 SNP sequence

GCACCCGCATGGGAGTAGATCGCCGATGCGGCTCCTTCAGCACTGGACTG[T/C]**GTAGTTTCACCGGTTGCACCCTCACTGCCTGGGGCGTACCCATAGTGCAT**

Figure 1. SNP flanking sequence initially used for design of probe Excalibur_c25522_755 for the 90k SNP array. The sequence was obtained from (Wang et al. 2014, Supplementary Table S5). The 50 bp probe sequence is the reverse complement of the sequence shown here in bold and can be identified using the Illumina manifest file, ILMN Strand = Design Strand (Supplementary Table S2).

ID	Primer_AlleleFAM	Primer_AlleleHEX	Primer_Common
Excalibur_c25522_755	GGGTGCAACCGGTGAAACTACA	GGTGCAACCGGTGAAACTACG	TAGATCGCCGATGCGGCTCCTT

Figure 2. Primers designed by LGC Biosearch Technologies using the "KASP by Design" service.

When we applied this KASP assay on our reference genotypes, the calls of genotypes were not consistent with the SNP chip data. This suggested a putative lack of locus-specificity of the designed KASP assay. For evaluation we performed a BLASTn analysis of the 101 bp SNP flanking sequence against the wheat reference genome IWGSC RefSeq v1.0 cv. Chinese Spring and also aligned the KASP primers. The alignment confirmed that the common KASP primer is not specific to the target on genome B but will also bind to two consecutive regions on the B, A and D genome homeologous regions (Figure 3).

```
                                    Common primer          Allele specific primers
IWGSC:5D             GGCACCAGCATGGGAGTAGATtGCCGATGCGGCtCtTTCAGCACTGGACTGCGTAGTTTC   60
IWGSC:5A             GGCACCAGCCTGGGAGTAGATtGCCGATGCaGCgCCTTCAGCACTGGACTGCGTAGTTTC   60
IWGSC:5B             GGCACCCGCATGGGAGTAGATCaCtGATGCaGCTCCTTCAGCACTGGACTGCGTAGTTTC   60

IWGSC:5D             tgCGGTTGCACCCTCGCTGCCTGGGGCATACCCATAGTGCATGACAAGTTGCAGGTTCGT 120
IWGSC:5A             tCCGGTTGCACCCTCGCTGCCTGGGGCGTACCCATAGTGCATGACAAGTTGCAGGTTTGT 120
IWGSC:5B             ttCGGTTGCACCATAGCTGCCTGGGGCGTACCCATAGTGCATGACAAGTTGCAGGTTTGT 120

IWGSC:5D             CCCGGCCACTTCATGTGGTGCAGCTGCGACGGCGTACACCAATGGCGCAGC--------- 171
IWGSC:5A             CCCGGCCACTTCATGTGGTGCAGCTGCGACGGCGTACACCAATGGCGCAGCCTGAGTGGT 180
IWGSC:5B             CACGGCCACGTCATGTGGTGCAGGTGCGATGGCGT------------------------  155

                                               Common primer
IWGSC:5D              --------------CATGGGAGTAGAcCGCCGATGCGGCTCCTTCAGCACTGGACTGCGT   217
IWGSC:5A              TGCTGGGGCACCCGCATGGGAGTAGATCGCCGATGCaGCTCCTTCAGCACTGGACTGTGT   240
IWGSC:5B              TGCTGGGGCACCCGCATGGGAGTAGATCGCCGATGCGGCTCCTTCAGCACTGGACTGCGT   215
Excalibur_c25522_755         GCACCCGCATGGGAGTAGATCGCCGATGCGGCTCCTTCAGCACTGGACTGYGT
                                                                               SNP
                    Allele specific primers
IWGSC:5D              gGTTTCtCCGGTTGCACaCTCGCTGCCTGGGGCGTACACACAATGCATGACAAGTTGCGG   277
IWGSC:5A              AGTTTCtCtGGTTGCACCCTCACTGCCTGGGGCGTACCCATAGTGCATGACAAGTTGgAG   300
IWGSC:5B              AGTTTCACCGGTTGCACCCTCACTGCCTGGGGCGTACCCATAGTGCATGACAAGTTGCAG   275
Excalibur_c25522_755  AGTTTCACCGGTTGCACCCTCACTGCCTGGGGCGTACCCATAGTGCAT
```

Figure 3. Alignment of Excalibur_c25522_755 SNP flanking and KASP primer sequences designed by LGC Bioserarch Technologies against the wheat reference genome IWGSC. Blue bold letters represent the allele specific primer binding site. Black bold letters represent the common primer binding site. Green color letters represent the Excalibur_c25522_755 SNP flanking sequences (101 bp) (the sequence used to design the SNP probe by Illumina).

2) Second approach

The wheat reference genome IWGSC RefSeq Chinese Spring v1.0 was used to improve locus-specificity of the KASP assay. A KASP assay specific for the SNP detected by probe Excalibur_c25222_755 was designed applying two different methods:

2A) using the online tool PolyMarker which uses the IWGSC reference and

2B) using a comparative alignment of the SNP flanking sequence to the IWSSC reference as shown in Figure 7 followed by visual placement of KASP primers

2A) KASP primers designed by the PolyMarker pipeline using the IWGSC reference

ID	Primer_AlleleFAM	Primer_AlleleHEX	Primer_Common
Excalibur_c25522_755	TCCTTCAGCACTGGACTGT	TCCTTCAGCACTGGACTGC	GCAACTTGTCATGCACTATGG

Figure 4. Output of PolyMarker at http://www.polymarker.info/

However, alignment of the KASP primer sequences suggested by PolyMarker to the IWGSC reference genome showed that the primers are not specific to the target region on chromosome 5B, but similarly to the KASP primers develop by LGC Biosearch Technologies these primers were binding two consecutive regions on chromosomes 5B, 5A and 5D (Figure 5).

```
                                                            Allele specific primers
IWGSC:5D              GGCACCAGCATGGGAGTAGATTGCCGATGCGGCtCTTTCAGCACTGGACTGCGTAGTTTC   60
IWGSC:5A              GGCACCAGCCTGGGAGTAGATTGCCGATGCAGCgCCTTCAGCACTGGACTGCGTAGTTTC   60
IWGSC:5B              GGCACCCGCATGGGAGTAGATCACTGATGCAGCTCCTTCAGCACTGGACTGCGTAGTTTC   60

                                                            Common primer
IWGSC:5D              TGCGGTTGCACCCTCGCTGCCTGGGGCATACCCATAGTGCATGACAAGTTGCAGGTTCGT   120
IWGSC:5A              TCCGGTTGCACCCTCGCTGCCTGGGGCGTACCCATAGTGCATGACAAGTTGCAGGTTTGT   120
IWGSC:5B              TTCGGTTGCACCATAGCTGCCTGGGGCGTACCCATAGTGCATGACAAGTTGCAGGTTTGT   120

IWGSC:5D              CCCGGCCACTTCATGTGGTGCAGCTGCGACGGCGTACACCAATGGCGCAGC---------   171
IWGSC:5A              CCCGGCCACTTCATGTGGTGCAGCTGCGACGGCGTACACCAATGGCGCAGCCTGAGTGGT   180
IWGSC:5B              CACGGCCACGTCATGTGGTGCAGGTGCGATGGCGT-------------------------   155

                                                                  Allele specific primers
IWGSC:5D               --------------CATGGGAGTAGACCGCCGATGCGGCTCCTTCAGCACTGGACTGCGT   217
IWGSC:5A               TGCTGGGGCACCCGCATGGGAGTAGATCGCCGATGCaGCTCCTTCAGCACTGGACTGTGT   240
IWGSC:5B               TGCTGGGGCACCCGCATGGGAGTAGATCGCCGATGCGGCTCCTTCAGCACTGGACTGCGT   215
Excalibur_c25522_755          GCACCCGCATGGGAGTAGATCGCCGATGCGGCTCCTTCAGCACTGGACTGYGT
                                                                                  SNP
                                                                   Common primer
IWGSC:5D               gGTTTCtCCGGTTGCACaCTCGCTGCCTGGGGCGTACACACAATGCATGACAAGTTGCGG   277
IWGSC:5A               AGTTTCtCTGGTTGCACCCTCACTGCCTGGGGCGTACCCATAGTGCATGACAAGTTGgAG   300
IWGSC:5B               AGTTTCACCGGTTGCACCCTCACTGCCTGGGGCGTACCCATAGTGCATGACAAGTTGCAG   275
Excalibur_c25522_755   AGTTTCACCGGTTGCACCCTCACTGCCTGGGGCGTACCCATAGTGCAT
```

Figure 5. Alignment of Excalibur_c25522_755 SNP flanking and KASP primer sequences designed by PolyMarker against the wheat reference genome IWGSC. Blue bold letters represent the allele specific primer binding site. Black bold letters represent the common primer binding site. Green color letters represent the Excalibur_c25522_755 SNP flanking sequences (101 bp) (the sequence used to design the SNP probe by Illumina).

2B) KASP primers designed by visual placement using the IWGSC reference

ID	Primer_AlleleFAM	Primer_AlleleHEX	Primer_Common
Excalibur_c25522_755	GGTGCAACCGGTGAAACTACA	GGTGCAACCGGTGAAACTACG	GTCATGTGGTGCAGGTGCGAT

Figure 6. KASP primers designed by visual placement from alignment in Figure 7.

The KASP primers were designed based on the obtained comparative alignment with the IWGSC RefSeq Chinese Spring v1.0 reference genome (Figure 7).

```
IWGSC:5D             GGTGACAGGGTAATCACAGGTTAGCCGGTGCAGCTGCCACGGCATACACCGATGGCACAG   60
IWGSC:5A             GGTGACAGGGCAATCACAGGTTAGCCGGTGCAGCTGCGACGGCATATACCGATGGCACAG   60
IWGSC:5B             GGTGACAGGGCAATCACAGGTTAGCCGGTGCAGCTGCCACGGCATACACCGATGGCGCAG   60

IWGSC:5D             CCTGAGTGGTCGCTGGGGCACCAGCATGGGAGTAGATTGCCGATGCGGCTCTTTCAGCAC  120
IWGSC:5A             CCTGAGTGGTCACTGGGGCACCAGCCTGGGAGTAGATTGCCGATGCAGCGCCTTCAGCAC  120
IWGSC:5B             CCTGAGTGGTCACTGGGGCACCCGCATGGGAGTAGATCACTGATGCAGCTCCTTCAGCAC  120

                            Allele specific primers
IWGSC:5D             TGGACTGCGTAGTTTCtgCGGTTGCACCCTCGCTGCCTGGGGCATACCCATAGTGCATGA  180
IWGSC:5A             TGGACTGCGTAGTTTCtCCGGTTGCACCCTCGCTGCCTGGGGCGTACCCATAGTGCATGA  180
IWGSC:5B             TGGACTGCGTAGTTTCttCGGTTGCACCATAGCTGCCTGGGGCGTACCCATAGTGCATGA  180

IWGSC:5D             CAAGTTGCAGGTTCGTCCCGGCCACTTCATGTGGTGCAGCTGCGACGGCGTACACCAATG  240
IWGSC:5A             CAAGTTGCAGGTTTGTCCCGGCCACTTCATGTGGTGCAGCTGCGACGGCGTACACCAATG  240
IWGSC:5B             CAAGTTGCAGGTTTGTCACGGCCACGTCATGTGGTGCAGGTGCGATGGCGT---------  231
                                                Common primer

IWGSC:5D             GCGCAGC-----------------------CATGGGAGTAGACCGCCGATGCGGCTCCTT  277
IWGSC:5A             GCGCAGCCTGAGTGGTTGCTGGGGCACCCGCATGGGAGTAGATCGCCGATGCAGCTCCTT  300
IWGSC:5B             ----------------TGCTGGGGCACCCGCATGGGAGTAGATCGCCGATGCGGCTCCTT  275
Excalibur_c25522_755                         GCACCCGCATGGGAGTAGATCGCCGATGCGGCTCCTT

                                Allele specific primers
IWGSC:5D             CAGCACTGGACTGCGTgGTTTCtCCGGTTGCACaCTCGCTGCCTGGGGCGTACACACAAT  337
IWGSC:5A             CAGCACTGGACTGTGTAGTTTCtCtGGTTGCACCCTCACTGCCTGGGGCGTACCCATAGT  360
IWGSC:5B             CAGCACTGGACTGCGTAGTTTCACCGGTTGCACCCTCACTGCCTGGGGCGTACCCATAGT  335
Excalibur_c25522_755 CAGCACTGGACTGYGTAGTTTCACCGGTTGCACCCTCACTGCCTGGGGCGTACCCATAGT
                                  SNP

IWGSC:5D             GCATGACAAGTTGCGGGTTCTTCGCGGCCACTTCATGTGGTGCAGCAGCTCTGAAACACA  397
IWGSC:5A             GCATGACAAGTTGGAGGTTTGTCGCGGCCACTTCATGTGGTGCAGCAGCTCTGAAACACA  420
IWGSC:5B             GCATGACAAGTTGCAGGTTCGTCGCGGCCACTTCATGTGGTGCAGCAGCTCTGAAACACA  395
Excalibur_c25522_755 GCAT
```

Figure 7. Alignment of Excalibur_c25522_755 SNP flanking and KASP primer sequences designed by visual placement against the wheat reference genome IWGSC. Blue bold letters represent the allele specific primer binding site. Black bold letters represent the common primer binding site. Green color letters represent the Excalibur_c25522_755 SNP flanking sequences (101 bp) (the sequence used to design the SNP probe by Illumina).

The KASP assay primers for Excalibur_c25522_755 SNP were redesigned to specifically only amplify the target region on chromosome 5B by making sure that the common primer did only anneal to one site on chromosome 5B. However, when these redesigned KASP primers were applied with the set of reference genotypes, the allelic discrimination plot showed that the calls of genotypes were not consistent with the SNP chip data indicating that the used reference IWGSC alone might not be sufficient to design locus-specific KASP assay.

3) Third approach

For this reason the KASP assay for probe Excalibur_c25522_755 was redesigned (Figure 8) based on alignment of multiple wheat genomic resources plus Sanger sequencing of flanking regions of selected genotypes from our reference set and visual placement of primers (Figure 9). The alignment confirmed that the IWGSC reference genome is insufficient to cover all the diversity in our wheat diversity panel, where multiple sequences alignment showed that the common primer which was visually designed in the second approach has a mismatch at 3 end of the primer binding site for some wheat genotypes (grey color box in Figure 9). In contrast, the redesigned KASP assay primers (Figure 8) using multiple aligned resources gave the expected SNP calls for our reference genotype set.

ID	Primer_AlleleFAM	Primer_AlleleHEX	Primer_Common
Excalibur_c25522_755	GGCTCCTTCAGCACTGGACTGT	GCTCCTTCAGCACTGGACTGC	CAGTGAGGGTGCAACCGGT

Figure 8. Primers designed by visual placement from multiple alignment in Figure 9.

```
                                                        Allele specific primers
IWGSC:5D          TCGCTGGGGCACCAGCATGGGAGTAGATTGCCGATGCGGCTCtTTCAGCACTGGACTGCG
IWGSC:5A          TCACTGGGGCACCAGCCTGGGAGTAGATTGCCGATGCAGCgCCTTCAGCACTGGACTGCG
419_5B            TCACTGGGGCACCCGCATGGGAGTAGATCACTGATGCAGCTCCTTCAGCACTGGACTGCG
126_5B            TCACTGGGGCACCCGCATGGGAGTAGATCACTGATGCAGCTCCTTCAGCACTGGACTGCG
311_5B            ------------CCGCATGGGAGTAGATCACTGATGCAGCTCCTTCAGCACTGGACTGCG
Paragon_5B        TCACTGGGGCACCCGCATGGGAGTAGATCACTGATGCAGCTCCTTCAGCACTGGACTGCG
Lancer_5B         TCACTGGGGCACCCGCATGGGAGTAGATCACTGATGCAGCTCCTTCAGCACTGGACTGCG
388_5B            ---------------CATGGGAGTAGATCACTGATGCAGCTCCTTCAGCACTGGACTGCG
378_5B            --------------GCATGGGAGTAGATCACTGATGCAGCTCCTTCAGCACTGGACTGCG
99_5B             -------------CGCATGGGAGTAGATCACTGATGCAGCTCCTTCAGCACTGGACTGCG
394_5B            TCACTGGGGCACCCGCATGGGAGTAGATCACTGATGCAGCTCCTTCAGCACTGGACTGCG
IWGSC_5B          TCACTGGGGCACCCGCATGGGAGTAGATCACTGATGCAGCTCCTTCAGCACTGGACTGCG
Julius_5B         TCACTGGGGCACCCGCATGGGAGTAGATCACTGATGCAGCTCCTTCAGCACTGGACTGCG
108_5B            TCACTGGGGCACCCGCATGGGAGTAGATCACTGATGCAGCTCCTTCAGCACTGGACTGCG
Jaggar_5B         TCACTGGGGCACCCGCATGGGAGTAGATCACTGATGCAGCTCCTTCAGCACTGGACTGCG
406_5B            TCACTGGGGCACCCGCATGGGAGTAGATCACTGATGCAGCTCCTTCAGCACTGGACTGCG
112_5B            TCACTGGGGCACCCGCATGGGAGTAGATCACTGATGCAGCTCCTTCAGCACTGGACTGCG
299_5B            TCACTGGGGCACCCGCATGGGAGTAGATCACTGATGCAGCTCCTTCAGCACTGGACTGCG
                                 * ***********  * ***** ** * *****************

IWGSC:5D          TAGTTTCTGCGGTTGCACCCTCGCTGCCTGGGGCATACCCATAGTGCATGACAAGTTGCA
IWGSC:5A          TAGTTTCTCCGGTTGCACCCTCGCTGCCTGGGGCGTACCCATAGTGCATGACAAGTTGCA
419_5B            TAGTTTCTTCGGTTGCACCCTAGCTGCCTGGGGCGTACCCGTAATGCATGACAAGTTGCA
```

```
126_5B               TAGTTTCTTCGGTTGCACCCTAGCTGCCTGGGGCGTACCCGTAATGCATGACAAGTTGCA
311_5B               TAGTTTCTTCGGTTGCACCCTAGCTGCCTGGGGCGTACCCGTAATGCATGACAAGTTGCA
Paragon_5B           TAGTTTCTTCGGTTGCACCCTAGCTGCCTGGGGCGTACCCGTAATGCATGACAAGTTGCA
Lancer_5B            TAGTTTCTTCGGTTGCACCCTAGCTGCCTGGGGCGTACCCGTAATGCATGACAAGTTGCA
388_5B               TAGTTTCTTCGGTTGCACCCTAGCTGCCTGGGGCGTACCCGTAATGCATGACAAGTTGCA
378_5B               TAGTTTCTTCGGTTGCACCCTAGCTGCCTGGGGCGTACCCGTAATGCATGACAAGTTGCA
99_5B                TAGTTTCTTCGGTTGCACCCTAGCTGCCTGGGGCGTACCCGTAATGCATGACAAGTTGCA
394_5B               TAGTTTCTTCGGTTGCACCATAGCTGCCTGGGGCGTACCCATAGTGCATGACAAGTTGCA
IWGSC_5B             TAGTTTCTTCGGTTGCACCATAGCTGCCTGGGGCGTACCCATAGTGCATGACAAGTTGCA
Julius_5B            TAGTTTCTTCGGTTGCACCATAGCTGCCTGGGGCGTACCCATAGTGCATGACAAGTTGCA
108_5B               TAGTTTCTTCGGTTGCACCATAGCTGCCTGGGGCGTACCCATAGTGCATGACAAGTTGCA
Jaggar_5B            TAGTTTCTTCGGTTGCACCATAGCTGCCTGGGGCGTACCCATAGTGCATGACAAGTTGCA
406_5B               TAGTTTCTTCGGTTGCACCATAGCTGCCTGGGGCGTACCCATAGTGCATGACAAGTTGCA
112_5B               TAGTTTCTTCGGTTGCACCATAGCTGCCTGGGGCGTACCCATAGTGCATGACAAGTTGCA
299_5B               TAGTTTCTTCGGTTGCACCATAGCTGCCTGGGGCGTACCCATAGTGCATGACAAGTTGCA
                     ******** ********** * ************ ***** ** ****************

IWGSC:5D             GGTTCGTCCCGGCCACTTCATGTGGTGCAGCTGCGACGGCGTACACCAATGGCGCAGC--
IWGSC:5A             GGTTTGTCCCGGCCACTTCATGTGGTGCAGCTGCGACGGCGTACACCAATGGCGCAGCCT
419_5B               GGTTCATCGCGGCCACGTCATGTGGTGCAGCTGCGACGGCGTACACCGATGGTGCAGACT
126_5B               GGTTCATCGCGGCCACGTCATGTGGTGCAGCTGCGACGGCGTACACCGATGGTGCAGACT
311_5B               GGTTCATCGCGGCCACGTCATGTGGTGCAGCTGCGACGGCGTACACCGATGGTGCAGACT
Paragon_5B           GGTTCATCGCGGCCACGTCATGTGGTGCAGCTGCGACGGCGTACACCGATGGTGCAGACT
Lancer_5B            GGTTCATCGCGGCCACGTCATGTGGTGCAGCTGCGACGGCGTACACCGATGGTGCAGACT
388_5B               GGTTCATCGCGGCCACGTCATGTGGTGCAGCTGCGACGGCGTACACCGATGGTGCAGACT
378_5B               GGTTCATCGCGGCCACGTCATGTGGTGCAGCTGCGACGGCGTACACCGATGGTGCAGACT
99_5B                GGTTCATCGCGGCCACGTCATGTGGTGCAGCTGCGACGGCGTACACCGATGGTGCAGACT
394_5B               GGTTTGTCACGGCCACGTCATGTGGTGCAGGTGCGACGGCGT------------------
IWGSC_5B             GGTTTGTCACGGCCACGTCATGTGGTGCAGGTGCGATGGCGT------------------
Julius_5B            GGTTTGTCACGGCCACGTCATGTGGTGCAGGTGCGATGGCGT------------------
108_5B               GGTTTGTCACGGCCACGTCATGTGGTGCAGGTGCGATGGCGT------------------
Jaggar_5B            GGTTTGTCACGGCCACGTCATGTGGTGCAGGTGCGACGGCGT------------------
406_5B               GGTTTGTCACGGCCACGTCATGTGGTGCAGGTGCGACGGCGT------------------
112_5B               GGTTTGTCACGGCCACGTCATGTGGTGCAGGTGCGACGGCGT------------------
299_5B               GGTTTGTCACGGCCACGTCATGTGGTGCAGGTGCGACGGCGT------------------
                     ****  ** ******* ************* ***** *****

                                                               Allele specific primers

IWGSC:5D             ---------------------CATGGGAGTAGACCGCCGATGCGGCTCCTTCAGCACTGG
IWGSC:5A             GAGTGGTTGCTGGGGCACCCGCATGGGAGTAGATCGCCGATGCaGCTCCTTCAGCACTGG
419_5B               GAGCGGTTGCTGGGGCAGCTGCATGGAAGTAGATCGCCGATGCGGCTCCTTCAGCACTGG
126_5B               GAGCGGTTGCTGGGGCAGCTGCATGGAAGTAGATCGCCGATGCGGCTCCTTCAGCACTGG
311_5B               GAGCGGTTGCTGGGGCAGCTGCATGGAAGTAGATCGCCGATGCGGCTCCTTCAGCACTGG
Paragon_5B           GAGCGGTTGCTGGGGCAGCTGCATGGAAGTAGATCGCCGATGCGGCTCCTTCAGCACTGG
Lancer_5B            GAGCGGTTGCTGGGGCAGCTGCATGGAAGTAGATCGCCGATGCGGCTCCTTCAGCACTGG
388_5B               GAGCGGTTGCTGGGGCAGCTGCATGGAAGTAGATCGCCGATGCGGCTCCTTCAGCACTGG
378_5B               GAGCGGTTGCTGGGGCAGCTGCATGGAAGTAGATCGCCGATGCGGCTCCTTCAGCACTGG
99_5B                GAGCGGTTGCTGGGGCAGCTGCATGGAAGTAGATCGCCGATGCGGCTCCTTCAGCACTGG
394_5B               -------TGCTGGGGCACCCGCATGGGAGTAGATCGCCGATGCGGCTCCTTCAGCACTGG
IWGSC_5B             -------TGCTGGGGCACCCGCATGGGAGTAGATCGCCGATGCGGCTCCTTCAGCACTGG
Julius_5B            -------TGCTGGGGCACCCGCATGGGAGTAGATCGCCGATGCGGCTCCTTCAGCACTGG
108_5B               -------TGCTGGGGCACCCGCATGGGAGTAGATCGCCGATGCGGCTCCTTCAGCACTGG
Jaggar_5B            -------TGCTGGGGCACCCGCATGGGAGTAGATCGCCGATGCGGCTCCTTCAGCACTGG
406_5B               -------TGCTGGGGCACCCGCATGGGAGTAGATCGCCGATGCGGCTCCTTCAGCACTGG
112_5B               -------TGCTGGGGCACCCGCATGGGAGTAGATCGCCGATGCGGCTCCTTCAGCACTGG
299_5B               -------TGCTGGGGCACCCGCATGGGAGTAGATCGCCGATGCGGCTCCTTCAGCACTGG
Excalibur_c25522_755               GCACCCGCATGGGAGTAGATCGCCGATGCGGCTCCTTCAGCACTGG
                                          ***** ****** ********* ****************
```

```
                                          Common primer
IWGSC:5D                 ACTGCGTGGTTTCTCCGGTTGCACACTCGCTGCCTGGGGCGTACACACAATGCATGACAA
IWGSC:5A                 ACTGTGTAGTTTCTCTGGTTGCACCCTCACTGCCTGGGGCGTACCCATAGTGCATGACAA
419_5B                   ACTGTGTAGTTTCACCGGTTGCACCCTCACTGCCTGGGGCATACCCATAGTGCATGACAA
126_5B                   ACTGTGTAGTTTCACCGGTTGCACCCTCACTGCCTGGGGCATACCCATAGTGCATGACAA
311_5B                   ACTGTGTAGTTTCACCGGTTGCACCCTCACTGCCTGGGGCATACCCATAGTGCATGACAA
Paragon_5B               ACTGTGTAGTTTCACCGGTTGCACCCTCACTGCCTGGGGCATACCCATAGTGCATGACAA
Lancer_5B                ACTGTGTAGTTTCACCGGTTGCACCCTCACTGCCTGGGGCATACCCATAGTGCATGACAA
388_5B                   ACTGTGTAGTTTCACCGGTTGCACCCTCACTGCCTGGGGCATACCCATAGTGCATGACAA
378_5B                   ACTGTGTAGTTTCACCGGTTGCACCCTCACTGCCTGGGGCATACCCATAGTGCATGACAA
99_5B                    ACTGTGTAGTTTCACCGGTTGCACCCTCACTGCCTGGGGCATACCCATAGTGCATGACAA
394_5B                   ACTGCGTAGTTTCACCGGTTGCACCCTCACTGCCTGGGGCGTACCCATAGTGCATGACAA
IWGSC_5B                 ACTGCGTAGTTTCACCGGTTGCACCCTCACTGCCTGGGGCGTACCCATAGTGCATGACAA
Julius_5B                ACTGCGTAGTTTCACCGGTTGCACCCTCACTGCCTGGGGCGTACCCATAGTGCATGACAA
108_5B                   ACTGCGTAGTTTCACCGGTTGCACCCTCACTGCCTGGGGCGTACCCATAGTGCATGACAA
Jaggar_5B                ACTGCGTAGTTTCACCGGTTGCACCCTCACTGCCTGGGGCGTACCCATAGTGCATGACAA
406_5B                   ACTGCGTAGTTTCACCGGTTGCACCCTCACTGCCTGGGGCGTACCCATAGTGCATGACAA
112_5B                   ACTGCGTAGTTTCACCGGTTGCACCCTCACTGCCTGGGGCGTACCCATAGTGCATGACAA
299_5B                   ACTGCGTAGTTTCACCGGTTGCACCCTCACTGCCTGGGGCGTACCCATAGTGCATGACAA
Excalibur_c25522_755     ACTGYGTAGTTTCACCGGTTGCACCCTCACTGCCTGGGGCGTACCCATAGTGCAT
                         **** ** ***** * ******** *** *********** *** ** * **********
                            SNP
```

Figure 9. Multiple wheat genomic resources plus Sanger sequencing of flanking regions of Excalibur_c25522_755 SNP. The numbers refer to wheat genotypes used in this study. Green color letters represent the Excalibur_c25522_755 SNP flanking sequences (101bp) (the sequence used to design the SNP probe by Illumina). The blue bold letters represent the allele specific primer binding site. Black bold letters represent the common primer binding site. The grey box represents the common primer binding site which designed by visual in the second approach using the reference genome IWGSC. The yellow color highlight letter represents the polymorphism between IWGSC sequence and the other genotypes.

Example 2: SNP probe Kukri_c46570_214 from haploblock Hap-5B-RDMa

1) First approach

101 bp flanking the SNP detected by probe Kukri_c46570_214 (Figure 10) were submitted according to the requirements to the company LGC Biosearch Technologies for their "KASP by Design". This service designs KASP primers without any further consideration of the wheat genome composition. The primer information is usually not released by the company. However, we received the primer information shown in Figure 2 for optimization of KASP assays,

Kukri_c46570_214 sequence

aggaaggagatcaacgaactcgcgcttaaaagatatcttcattttccagc[A/G]**CGCTTCGGATTCCTGGAGCGTTG CAAGATCACGCTCAAGTCCGTGGAGAG**

Figure 10. SNP flanking sequence initially used for design of probe Kukri_c46570_214 for the 90k SNP array. The sequence was obtained from (Wang et al. 2014, Supplementary Table S5). The 50 bp probe sequence is the reverse complement of the sequence shown here in bold and can be identified using the Illumina manifest file, ILMN Strand = Design Strand (Supplementary Table S2).

ID	Primer_AlleleFAM	Primer_AlleleHEX	Primer_Common
Kukri_c46570_214	CGCTTAAAAGATATCTTCATTTTCCAGCA	GCTTAAAAGATATCTTCATTTTCCAGCG	CTTGCAACGCTCCAGGAATCCGAA

Figure 11 Primers designed by LGC Biosearch Technologies using the “KASP by Design” service.

When we applied this KASP assay on our reference genotypes, the allelic discrimination plot showed no signal for any of the genotypes (Figure 12). For evaluation we performed a BLASTn analysis of the 101 bp SNP flanking sequence against the wheat reference genome IWGSC RefSeq v1.0 cv. Chinese Spring and also aligned the KASP primers. The alignment showed that the designed allele specific primers were aligning across exon-intron boundaries within the wheat genome, consequently leading to a failure of the KASP assays (Figure 13).

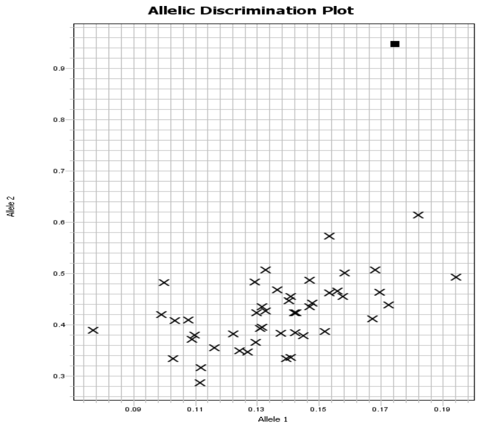

Figure 12. Discrimination plot for KASP assay designed LGC Biosearch Technologies using the ”KASP by Design” service from SNP flanking sequence Kukri_c46570_214

```
IWGSC:5A        TCGCAGGCTTGGTTAAGGAAGGAGATCAACGAACTCGCGCTTAAAAGATATCTTGTAAGT 60
IWGSC:5B1       TCACAGGCTTGGTTAAGGAAGGAGATCAACGGACTCGCGCTTAAAAGATATCTTGTAAGT 60
IWGSC:5B2       TCGCAGGCTTGGTTAAGGAAGGAGATCAACGAACTCGCGCTTAAAAGATATCTTGTAAGT 60
IWGSC:5D        TCGCAGGCTTGGTTAAGGAAGGAGATCAACGAACTCGCGCTTAAAAGATATCTTGTAAGT 60
Kukri_c46570_214               aggaaggagatcaacgaactcgcgcttaaaagatatctt
                ** **************************** ****************************

IWGSC:5A        ACTTGACATGCCAGTTTATTATCA-ACTGAGCAAATTAGGAACTTGTTTGAAAAAAGAAA 120
IWGSC:5B1       AC-----------AATCATTATTT--------------------------GAAAAAGAGA 83
IWGSC:5B2       AC-----------AATCATTATTT--------------------------GAAAAAGAGA 83
IWGSC:5D        AC-----------AATCATTATTTTACTCTGCAAATTAGGAACTTGTTTGAAAAAAGAGA 109
                **             * *****                         ******* *

IWGSC:5A        GTAAGAAATAAGAGTGCACCATTTGGAAATAAGGGAACCAACCAACCAATCAAACTACTT 180
IWGSC:5B1       GTAAGAAATAAGAGTGCACCTTTTGGAAATAAGGGAACCAACCAACCAATCAAACTACTT 143
IWGSC:5B2       GTAAGAAATAAGAGTGCACCTTTTGGAAATAAGGGAACCAACCAACCAATCAAACTACTT 143
IWGSC:5D        GTAAGAAATAAGAGTGCACCTTTTGGAAATAAGGGAACCAACCAACCAATCAAACTACTT 169
                ******************** ***************************************

IWGSC:5A        GACATGCCAGTGTATTATCAACTCGGTGCATAATATTTGTCATC--ATACACCCACGAAC 238
```

```
IWGSC:5B1           GACATGCCAGTTTATTATCAACTAGGTGTATAATATTTGTCATC--ATACACCCACGAAC 201
IWGSC:5B2           GACATGCCAGTtTATTATCAACTCGGTGCATAATATTTGTCATC--ATACACCCACGAAC 201
IWGSC:5D            GACATGCCAGTGTATTATCAACTCGGTGCATAATATTTGTCATCATATACACCCACGAAC 229
                    *********** *********** **** ***************  **************

                                                          Allele specific primers

IWGSC:5A            TGTGATACAAATAACCCGTGCGATCGCAATGGCTTGCAGCATTTTCCAGCGCGCTTtGGA 298
IWGSC:5B1           TGTGATACAAATAACCCGTGCGATCGCAATGGCTTGCAGCATTTTCCAGCACGCTTCGGA 261
IWGSC:5B2           TGTGATACAAATAACCCGTGCGATCGCAATGGCTTGCAGCATTTTCCAGCGCGCTTCGGA 261
IWGSC:5D            TGTGATACAAATAACCCGTGCGATCGCAATGGCTTGCAGCATTTTCCAGCGCGCTTCGGA 289
Kukri_c46570_214                                           cattttccagcRCGCTTCGGA
                    ************************************************** ***** ***
                                                                      SNP
    Common primer

IWGSC:5A            TTCCTGGAGCGTTGCAAGATCACGCTCAAGTCCGTGGAGAGGAACAAGTCTTGGGAGGTG 358
IWGSC:5B1           TTCCTGGAGCGTTGCAAGATCACGCTCAAGTCCGTGGAGAGGAACAAGTCTTGGGAGGTG 321
IWGSC:5B2           TTCCTGGAGCGTTGCAAGATCACGCTCAAGTCCGTGGAGAGGAACAAGTCTTGGGAGGTG 321
IWGSC:5D            TTCCTGGAGCGTTGCAAGATCACGCTCAAGTCCGTGGAGAGGAACAAGTCTTGGGAGGTG 349
Kukri_c46570_214    TTCCTGGAGCGTTGCAAGATCACGCTCAAGTCCGTGGAGAG
                    ************************************************************

IWGSC:5A            GAAGGTGTCAACTACCATCCAGGCACCAGCAGGTCCTATAATTGTCTTACGAGAGGCTGG 418
IWGSC:5B1           GAAGGTGTCAACTACCATCCAGGCACCAACAGGTCTTATAATTGTCTTACGAGAGGCTGG 381
IWGSC:5B2           GAAGG-------------------------------------TGTCTTACGAGAGGCTGG 344
IWGSC:5D            GAAGGTGTCAACTACCATCCAGGCACCCGCAGGTCTTATAATTGTCTTACGAGAGGCTGG 409
                    *****                                     ******************

IWGSC:5A            AAGGCCTTCTGTAAGGAGAACGAGCTCAAGGCAGGAGACATCTGCACCTTCAAAGTCGTC 478
IWGSC:5B1           AAGGCCTTCTGTAAGGAGAACGAGCTCAAGGCAGGAGACGTCTGCACCTTCAAAGTCATC 441
IWGSC:5B2           AAGGCCTTCTGTAAGGAGAACGAGCTCAAGGCAGGAGACATCTGCACCTTCAAAGTCGTC 404
IWGSC:5D            AAGGCCTTCTGTAAGGAGAACGAGCTCAAGGCAGGAGACATCTGCACCTTCAAAGTCGTC 469
                    *************************************** ***************** **
```

Figure 13. Alignment of Kukri_c46570_214 SNP flanking and KASP primer sequences designed by LGC Bioserarch Technologies against the wheat reference genome IWGSC. Blue bold letters represent the allele specific primer binding site. Black bold letters represent the common primer binding site. Green color letters represent the Kukri_c46570_214 SNP flanking sequences (101 bp) (the sequence used to design the SNP probe by Illumina). Allele-specific primer binding site contains an intron of 186 bp. The two primer parts were aligned to two neighboring exons (in boxes). Red lowercase letter represents one mismatch (G:A) between the probe sequence provide by Illumina and the chromosome 5B1 sequence.

2) Second approach

The wheat reference genome IWGSC RefSeq Chinese Spring v1.0 was used to improve locus-specificity of the KASP assay. A KASP assay specific for the SNP detected by probe Kukri_c46570_214 was designed applying two different methods:

2A) using the online tool PolyMarker which uses the IWGSC reference and

2B) using comparative alignment of the SNP flanking sequence to the IWSSC reference as shown in Figure 17 followed by visual placement of KASP primers

2A) KASP primers designed by the PolyMarker pipeline using the IWGSC reference

ID	Primer_AlleleFAM	Primer_AlleleHEX	Primer_Common
Kukri_c46570_214	TCCAGGAATCCGAAGCGT	TCCAGGAATCCGAAGCGC	CAGTGTATTATCAACTCGGTGC

Figure 14. Output of PolyMarker at http://www.polymarker.info/

Alignment of the KASP primer sequences suggested by PolyMarker to the IWGSC reference genome showed that the primers are not specific to the target region on chromosome 5B, but instead were binding to chromosomes 5A, 5B and 5D (Figure 15).

```
IWGSC:5A          TCGCAGGCTTGGTTAAGGAAGGAGATCAACGAACTCGCGCTTAAAAGATATCTTGTAAGT 60
IWGSC:5B1         TCACAGGCTTGGTTAAGGAAGGAGATCAACGgACTCGCGCTTAAAAGATATCTTGTAAGT 60
IWGSC:5B2         TCGCAGGCTTGGTTAAGGAAGGAGATCAACGAACTCGCGCTTAAAAGATATCTTGTAAGT 60
IWGSC:5D          TCGCAGGCTTGGTTAAGGAAGGAGATCAACGAACTCGCGCTTAAAAGATATCTTGTAAGT 60
Kukri_c46570_214                 aggaaggagatcaacgaactcgcgcttaaaagatatctt
                  ** **************************** ****************************

IWGSC:5A          ACTTGACATGCCAGTTTATTATCA-ACTGAGCAAATTAGGAACTTGTTTGAAAAAAGAAA 120
IWGSC:5B1         AC-----------AATCATTATT---------------------------GAAAAAGAGA 83
IWGSC:5B2         AC-----------AATCATTATT---------------------------GAAAAAGAGA 83
IWGSC:5D          AC-----------AATCATTATTTACTCTGCAAATTAGGAACTTGTTTGAAAAAAGAGA 109
                  **             * *****                             ******* *

IWGSC:5A          GTAAGAAATAAGAGTGCACCATTTGGAAATAAGGGAACCAACCAACCAATCAAACTACTT 180
IWGSC:5B1         GTAAGAAATAAGAGTGCACCTTTTGGAAATAAGGGAACCAACCAACCAATCAAACTACTT 143
IWGSC:5B2         GTAAGAAATAAGAGTGCACCTTTTGGAAATAAGGGAACCAACCAACCAATCAAACTACTT 143
IWGSC:5D          GTAAGAAATAAGAGTGCACCTTTTGGAAATAAGGGAACCAACCAACCAATCAAACTACTT 169
                  ******************** ***************************************
                            Common primer
IWGSC:5A          GACATGCCAGTGTATTATCAACTCGGTGCATAATATTTGTCATC--ATACACCCACGAAC 238
IWGSC:5B1         GACATGCCAGTTTATTATCAACTAGGTGTATAATATTTGTCATC--ATACACCCACGAAC 201
IWGSC:5B2         GACATGCCAGTtTATTATCAACTCGGTGCATAATATTTGTCATC--ATACACCCACGAAC 201
IWGSC:5D          GACATGCCAGTGTATTATCAACTCGGTGCATAATATTTGTCATCATATACACCCACGAAC 229
                  *********** *********** **** ***************  **************

                                                          Allele specific primers
IWGSC:5A          TGTGATACAAATAACCCGTGCGATCGCAATGGCTTGCAGCATTTTCCAGCGCGCTTtGGA 298
IWGSC:5B1         TGTGATACAAATAACCCGTGCGATCGCAATGGCTTGCAGCATTTTCCAGCACGCTTCGGA 261
IWGSC:5B2         TGTGATACAAATAACCCGTGCGATCGCAATGGCTTGCAGCATTTTCCAGCGCGCTTCGGA 261
IWGSC:5D          TGTGATACAAATAACCCGTGCGATCGCAATGGCTTGCAGCATTTTCCAGCGCGCTTCGGA 289
Kukri_c46570_214                                         cattttccagcRCGCTTCGGA
                  ************************************************** ***** ***
                                                                    SNP

IWGSC:5A          TTCCTGGAGCGTTGCAAGATCACGCTCAAGTCCGTGGAGAGGAACAAGTCTTGGGAGGTG 358
IWGSC:5B1         TTCCTGGAGCGTTGCAAGATCACGCTCAAGTCCGTGGAGAGGAACAAGTCTTGGGAGGTG 321
IWGSC:5B2         TTCCTGGAGCGTTGCAAGATCACGCTCAAGTCCGTGGAGAGGAACAAGTCTTGGGAGGTG 321
IWGSC:5D          TTCCTGGAGCGTTGCAAGATCACGCTCAAGTCCGTGGAGAGGAACAAGTCTTGGGAGGTG 349
Kukri_c46570_214  TTCCTGGAGCGTTGCAAGATCACGCTCAAGTCCGTGGAGAG
                  ************************************************************
```

```
IWGSC:5A        GAAGGTGTCAACTACCATCCAGGCACCAGCAGGTCCTATAATTGTCTTACGAGAGGCTGG 418
IWGSC:5B1       GAAGGTGTCAACTACCATCCAGGCACCAACAGGTCTTATAATTGTCTTACGAGAGGCTGG 381
IWGSC:5B2       GAAGG-------------------------------------TGTCTTACGAGAGGCTGG 344
IWGSC:5D        GAAGGTGTCAACTACCATCCAGGCACCCGCAGGTCTTATAATTGTCTTACGAGAGGCTGG 409
                *****                                     ******************

IWGSC:5A        AAGGCCTTCTGTAAGGAGAACGAGCTCAAGGCAGGAGACATCTGCACCTTCAAAGTCGTC 478
IWGSC:5B1       AAGGCCTTCTGTAAGGAGAACGAGCTCAAGGCAGGAGACGTCTGCACCTTCAAAGTCATC 441
IWGSC:5B2       AAGGCCTTCTGTAAGGAGAACGAGCTCAAGGCAGGAGACATCTGCACCTTCAAAGTCGTC 404
IWGSC:5D        AAGGCCTTCTGTAAGGAGAACGAGCTCAAGGCAGGAGACATCTGCACCTTCAAAGTCGTC 469
                *************************************** ***************** **
```

Figure 15. Alignment of Kukri_c46570_214 SNP flanking and KASP primer sequences designed by PolyMarker against the wheat reference genome IWGSC. Blue bold letters represent the allele specific primer binding site. Black bold letters represent the common primer binding site. Green color letters represent the Kukri_c46570_214 flanking sequences (101 bp) (the sequence used to design the SNP probe by Illumina).

2B) KASP primers designed by visual placement using the IWGSC reference

Before designing the KASP assay, it is important to accurately identify the right sequence which carries the Kukri_c46570_214 SNP, because the BLASTn hits against IWGSC sequences showed that there are two hits with high identity on the 5B chromosome named 5B1 and 5B2 (Supplementary Table S4) being far apart from each other (about 55 kbp). The 5B2 sequence is 100% identical to the SNP-containing sequence (101 bp) provided by Illumina while the 5B1 sequence has one single mismatch (G:A) (small red color letter in see Figure 13). The 5B1 sequence was chosen to design KASP primers due to two reasons, firstly an exon overlaps the genomic location of the 5B1 sequence whereas the 5B2 sequence does not contain exons (90K SNP chip are derived from RNA-Seq data). Secondly, the reference genome IWGSC (Chinese spring variety) exhibits the haplotype Hap-5B-RDMa-h1 which means that it has to carry the allele A at the SNP site (5B1sequence carries the allele A while 5B2 sequence carries allele G at the SNP location). Later we confirmed this result by aligning multiple wheat genomic resources plus Sanger sequencing of flanking region of putative SNP on the 5B1 strand using genotypes displaying the two alleles at the Kukri_c46570_214 SNP site as also shown in Figure 18. After identifying the correct sequence which should be used to design a KASP assay, the KASP primers were designed based on the obtained comparative alignment with the IWGSC RefSeq Chinese Spring v1.0 reference genome by visual placement (Figure 17). The redesigned KASP assay following this approach gave the expected SNP calls for our reference genotype set and this was also confirmed by Sanger sequencing of selected genotypes (Figure 18).

ID	Primer_AlleleFAM	Primer_AlleleHEX	Primer_Common
Kukri_c46570_214 SNP	CTTGCAGCATTTTCCAGCG	GCTTGCAGCATTTTCCAGCA	CCTCTCGTAAGACAATTATAAGACCTGT

Figure 16. KASP primers designed by visual placement from alignment in Figure 17.

```
IWGSC:5A          TCGCAGGCTTGGTTAAGGAAGGAGATCAACGAACTCGCGCTTAAAAGATATCTTGTAAGT 60
IWGSC:5B1         TCACAGGCTTGGTTAAGGAAGGAGATCAACGgACTCGCGCTTAAAAGATATCTTGTAAGT 60
IWGSC:5B2         TCGCAGGCTTGGTTAAGGAAGGAGATCAACGAACTCGCGCTTAAAAGATATCTTGTAAGT 60
IWGSC:5D          TCGCAGGCTTGGTTAAGGAAGGAGATCAACGAACTCGCGCTTAAAAGATATCTTGTAAGT 60
Kukri_c46570_214                 aggaaggagatcaacgaactcgcgcttaaaagatatctt
                  ** **************************** ****************************

IWGSC:5A          ACTTGACATGCCAGTTTATTATCA-ACTGAGCAAATTAGGAACTTGTTTGAAAAAAGAAA 120
IWGSC:5B1         AC-----------AATCATTATTT---------------------------GAAAAAGAGA 83
IWGSC:5B2         AC-----------AATCATTATTT---------------------------GAAAAAGAGA 83
IWGSC:5D          AC-----------AATCATTATTTTACTCTGCAAATTAGGAACTTGTTTGAAAAAAGAGA 109
                  **             * *****                             ******* *

IWGSC:5A          GTAAGAAATAAGAGTGCACCATTTGGAAATAAGGGAACCAACCAACCAATCAAACTACTT 180
IWGSC:5B1         GTAAGAAATAAGAGTGCACCTTTTGGAAATAAGGGAACCAACCAACCAATCAAACTACTT 143
IWGSC:5B2         GTAAGAAATAAGAGTGCACCTTTTGGAAATAAGGGAACCAACCAACCAATCAAACTACTT 143
IWGSC:5D          GTAAGAAATAAGAGTGCACCTTTTGGAAATAAGGGAACCAACCAACCAATCAAACTACTT 169
                  ******************** ***************************************

IWGSC:5A          GACATGCCAGTGTATTATCAACTCGGTGCATAATATTTGTCATC--ATACACCCACGAAC 238
IWGSC:5B1         GACATGCCAGTTTATTATCAACTAGGTGTATAATATTTGTCATC--ATACACCCACGAAC 201
IWGSC:5B2         GACATGCCAGTtTATTATCAACTCGGTGCATAATATTTGTCATC--ATACACCCACGAAC 201
IWGSC:5D          GACATGCCAGTGTATTATCAACTCGGTGCATAATATTTGTCATCATATACACCCACGAAC 229
                  *********** *********** **** ***************  **************

                                               Allele specific primers
IWGSC:5A          TGTGATACAAATAACCCGTGCGATCGCAATGGCTTGCAGCATTTTCCAGCGCGCTTtGGA 298
IWGSC:5B1         TGTGATACAAATAACCCGTGCGATCGCAATGGCTTGCAGCATTTTCCAGCACGCTTCGGA 261
IWGSC:5B2         TGTGATACAAATAACCCGTGCGATCGCAATGGCTTGCAGCATTTTCCAGCGCGCTTCGGA 261
IWGSC:5D          TGTGATACAAATAACCCGTGCGATCGCAATGGCTTGCAGCATTTTCCAGCGCGCTTCGGA 289
Kukri_c46570_214                                         cattttccagcRCGCTTCGGA
                  ************************************************** ***** ***
                                                                  SNP

IWGSC:5A          TTCCTGGAGCGTTGCAAGATCACGCTCAAGTCCGTGGAGAGGAACAAGTCTTGGGAGGTG 358
IWGSC:5B1         TTCCTGGAGCGTTGCAAGATCACGCTCAAGTCCGTGGAGAGGAACAAGTCTTGGGAGGTG 321
IWGSC:5B2         TTCCTGGAGCGTTGCAAGATCACGCTCAAGTCCGTGGAGAGGAACAAGTCTTGGGAGGTG 321
IWGSC:5D          TTCCTGGAGCGTTGCAAGATCACGCTCAAGTCCGTGGAGAGGAACAAGTCTTGGGAGGTG 349
Kukri_c46570_214  TTCCTGGAGCGTTGCAAGATCACGCTCAAGTCCGTGGAGAG
                  ************************************************************
                                                   Common primer
IWGSC:5A          GAAGGTGTCAACTACCATCCAGGCACCAGCAGGTCCTATAATTGTCTTACGAGAGGCTGG 418
IWGSC:5B1         GAAGGTGTCAACTACCATCCAGGCACCAACAGGTCTTATAATTGTCTTACGAGAGGCTGG 381
IWGSC:5B2         GAAGG-------------------------------------TGTCTTACGAGAGGCTGG 344
IWGSC:5D          GAAGGTGTCAACTACCATCCAGGCACCCGCAGGTCTTATAATTGTCTTACGAGAGGCTGG 409
                  *****                                     ******************

IWGSC:5A          AAGGCCTTCTGTAAGGAGAACGAGCTCAAGGCAGGAGACATCTGCACCTTCAAAGTCGTC 478
IWGSC:5B1         AAGGCCTTCTGTAAGGAGAACGAGCTCAAGGCAGGAGACGTCTGCACCTTCAAAGTCATC 441
IWGSC:5B2         AAGGCCTTCTGTAAGGAGAACGAGCTCAAGGCAGGAGACATCTGCACCTTCAAAGTCGTC 404
IWGSC:5D          AAGGCCTTCTGTAAGGAGAACGAGCTCAAGGCAGGAGACATCTGCACCTTCAAAGTCGTC 469
                  *************************************** ***************** **
```

Figure 17. Alignment of Kukri_c46570_214 SNP flanking and KASP primer sequences designed by visual placement against the wheat reference genome IWGSC. Blue bold letters represent the allele specific primer binding site. Black bold letters represent the common primer binding site. Green color letters represent the Kukri_c46570_214 SNP flanking sequences (101 bp) (the sequence used to design the SNP probe by Illumina).

```
378H2-5B1         ----------------------------------------------ATCATACACCCACG
311H2-5B1         ----------------------------------------------ATCATACACCCACG
99H2-5B1          ----------------------------------------------ATCATACACCCACG
419H2-5B1         ----------------------------------------------ATCATACACCCACG
388H2-5B1         ----------------------------------------------ATCATACACCCACG
Lancer_5B1        TACTTGACATGCCAGTGTATTATCAACTCGGTGCATAATATTTGTCATCATACACCCACG
Paragon_5B1       TACTTGACATGCCAGTGTATTATCAACTCGGTGCATAATATTTGTCATCATACACCCACG
547H1-5B1         TACTTGACATGCCAGTTTATTATCAACTAGGTGTATAATATTTGTCATCATACACCCACG
julius_5B1        TACTTGACATGCCAGTTTATTATCAACTAGGTGTATAATATTTGTCATCATACACCCACG
IWGSC_5B1         TACTTGACATGCCAGTTTATTATCAACTAGGTGTATAATATTTGTCATCATACACCCACG
907H1-5B1         TACTTGACATGCCAGTTTATTATCAACTAGGTGTATAATATTTGTCATCATACACCCACG
314H1-5B1         TACTTGACATGCCAGTTTATTATCAACTAGGTGTATAATATTTGTCATCATACACCCACG
151H1-5B1         TACTTGACATGCCAGTTTATTATCAACTAGGTGTATAATATTTGTCATCATACACCCACG
604H1-5B1         TACTTGACATGCCAGTTTATTATCAACTAGGTGTATAATATTTGTCATCATACACCCACG
139H1-5B1         TACTTGACATGCCAGTTTATTATCAACTAGGTGTATAATATTTGTCATCATACACCCACG
61H1-5B1          TACTTGACATGCCAGTTTATTATCAACTAGGTGTATAATATTTGTCATCATACACCCACG
70H1-5B1          TACTTGACATGCCAGTTTATTATCAACTAGGTGTATAATATTTGTCATCATACACCCACG
634H1-5B1         TACTTGACATGCCAGTTTATTATCAACTAGGTGTATAATATTTGTCATCATACACCCACG
                                                                **************

378H2-5B1         AACTGTGATGCAAATAACCCGTGCGATCGCAATGGCTTGCAGCATTTTCCAGCGCGCTTT
311H2-5B1         AACTGTGATGCAAATAACCCGTGCGATCGCAATGGCTTGCAGCATTTTCCAGCGCGCTTT
99H2-5B1          AACTGTGATGCAAATAACCCGTGCGATCGCAATGGCTTGCAGCATTTTCCAGCGCGCTTT
419H2-5B1         AACTGTGATGCAAATAACCCGTGCGATCGCAATGGCTTGCAGCATTTTCCAGCGCGCTTT
388H2-5B1         AACTGTGATGCAAATAACCCGTGCGATCGCAATGGCTTGCAGCATTTTCCAGCGCGCTTT
Lancer_5B1        AACTGTGATGCAAATAACCCGTGCGATCGCAATGGCTTGCAGCATTTTCCAGCGCGCTTT
Paragon_5B1       AACTGTGATGCAAATAACCCGTGCGATCGCAATGGCTTGCAGCATTTTCCAGCGCGCTTT
547H1-5B1         AACTGTGATACAAATAACCCGTGCGATCGCAATGGCTTGCAGCATTTTCCAGCACGCTTC
julius_5B1        AACTGTGATACAAATAACCCGTGCGATCGCAATGGCTTGCAGCATTTTCCAGCACGCTTC
IWGSC_5B1         AACTGTGATACAAATAACCCGTGCGATCGCAATGGCTTGCAGCATTTTCCAGCACGCTTC
907H1-5B1         AACTGTGATACAAATAACCCGTGCGATCGCAATGGCTTGCAGCATTTTCCAGCACGCTTC
314H1-5B1         AACTGTGATACAAATAACCCGTGCGATCGCAATGGCTTGCAGCATTTTCCAGCACGCTTC
151H1-5B1         AACTGTGATACAAATAACCCGTGCGATCGCAATGGCTTGCAGCATTTTCCAGCACGCTTC
604H1-5B1         AACTGTGATACAAATAACCCGTGCGATCGCAATGGCTTGCAGCATTTTCCAGCACGCTTC
139H1-5B1         AACTGTGATACAAATAACCCGTGCGATCGCAATGGCTTGCAGCATTTTCCAGCACGCTTC
61H1-5B1          AACTGTGATACAAATAACCCGTGCGATCGCAATGGCTTGCAGCATTTTCCAGCACGCTTC
70H1-5B1          AACTGTGATACAAATAACCCGTGCGATCGCAATGGCTTGCAGCATTTTCCAGCACGCTTC
634H1-5B1         AACTGTGATACAAATAACCCGTGCGATCGCAATGGCTTGCAGCATTTTCCAGCACGCTTC
                  ********* ******************************************* ***** 
                                                                     SNP

378H2-5B1         GGATTCCTGGAGCGTTGCAAGATCACGCTCAAGTCCGTGGAGAGGAACAAGTCTTGGGAG
311H2-5B1         GGATTCCTGGAGCGTTGCAAGATCACGCTCAAGTCCGTGGAGAGGAACAAGTCTTGGGAG
99H2-5B1          GGATTCCTGGAGCGTTGCAAGATCACGCTCAAGTCCGTGGAGAGGAACAAGTCTTGGGAG
419H2-5B1         GGATTCCTGGAGCGTTGCAAGATCACGCTCAAGTCCGTGGAGAGGAACAAGTCTTGGGAG
388H2-5B1         GGATTCCTGGAGCGTTGCAAGATCACGCTCAAGTCCGTGGAGAGGAACAAGTCTTGGGAG
Lancer_5B1        GGATTCCTGGAGCGTTGCAAGATCACGCTCAAGTCCGTGGAGAGGAACAAGTCTTGGGAG
Paragon_5B1       GGATTCCTGGAGCGTTGCAAGATCACGCTCAAGTCCGTGGAGAGGAACAAGTCTTGGGAG
547H1-5B1         GGATTCCTGGAGCGTTGCAAGATCACGCTCAAGTCCGTGGAGAGGAACAAGTCTTGGGAG
julius_5B1        GGATTCCTGGAGCGTTGCAAGATCACGCTCAAGTCCGTGGAGAGGAACAAGTCTTGGGAG
IWGSC_5B1         GGATTCCTGGAGCGTTGCAAGATCACGCTCAAGTCCGTGGAGAGGAACAAGTCTTGGGAG
907H1-5B1         GGATTCCTGGAGCGTTGCAAGATCACGCTCAAGTCCGTGGAGAGGAACAAGTCTTGGGAG
314H1-5B1         GGATTCCTGGAGCGTTGCAAGATCACGCTCAAGTCCGTGGAGAGGAACAAGTCTTGGGAG
151H1-5B1         GGATTCCTGGAGCGTTGCAAGATCACGCTCAAGTCCGTGGAGAGGAACAAGTCTTGGGAG
604H1-5B1         GGATTCCTGGAGCGTTGCAAGATCACGCTCAAGTCCGTGGAGAGGAACAAGTCTTGGGAG
139H1-5B1         GGATTCCTGGAGCGTTGCAAGATCACGCTCAAGTCCGTGGAGAGGAACAAGTCTTGGGAG
61H1-5B1          GGATTCCTGGAGCGTTGCAAGATCACGCTCAAGTCCGTGGAGAGGAACAAGTCTTGGGAG
70H1-5B1          GGATTCCTGGAGCGTTGCAAGATCACGCTCAAGTCCGTGGAGAGGAACAAGTCTTGGGAG
634H1-5B1         GGATTCCTGGAGCGTTGCAAGATCACGCTCAAGTCCGTGGAGAGGAACAAGTCTTGGGAG
                  ************************************************************
```

```
378H2-5B1         GTGGAAGGTGTCAACTACCATCCAGGCACCAACAGGTCTTATAATTGTCTTACGAGAGGC
311H2-5B1         GTGGAAGGTGTCAACTACCATCCAGGCACCAACAGGTCTTATAATTGTCTTACGAGAGGC
99H2-5B1          GTGGAAGGTGTCAACTACCATCCAGGCACCAACAGGTCTTATAATTGTCTTACGAGAGGC
419H2-5B1         GTGGAAGGTGTCAACTACCATCCAGGCACCAACAGGTCTTATAATTGTCTTACGAGAGGC
388H2-5B1         GTGGAAGGTGTCAACTACCATCCAGGCACCAACAGGTCTTATAATTGTCTTACGAGAGGC
Lancer_5B1        GTGGAAGGTGTCAACTACCATCCAGGCACCAACAGGTCTTATAATTGTCTTACGAGAGGC
Paragon_5B1       GTGGAAGGTGTCAACTACCATCCAGGCACCAACAGGTCTTATAATTGTCTTACGAGAGGC
547H1-5B1         GTGGAAGGTGTCAACTACCATCCAGGCACCAACAGGTCTTATAATTGTCTTACGAGAGGC
julius_5B1        GTGGAAGGTGTCAACTACCATCCAGGCACCAACAGGTCTTATAATTGTCTTACGAGAGGC
IWGSC_5B1         GTGGAAGGTGTCAACTACCATCCAGGCACCAACAGGTCTTATAATTGTCTTACGAGAGGC
907H1-5B1         GTGGAAGGTGTCAACTACCATCCAGGCACCAACAGGTCTTATAATTGTCTTACGAGAGGC
314H1-5B1         GTGGAAGGTGTCAACTACCATCCAGGCACCAACAGGTCTTATAATTGTCTTACGAGAGGC
151H1-5B1         GTGGAAGGTGTCAACTACCATCCAGGCACCAACAGGTCTTATAATTGTCTTACGAGAGGC
604H1-5B1         GTGGAAGGTGTCAACTACCATCCAGGCACCAACAGGTCTTATAATTGTCTTACGAGAGGC
139H1-5B1         GTGGAAGGTGTCAACTACCATCCAGGCACCAACAGGTCTTATAATTGTCTTACGAGAGGC
61H1-5B1          GTGGAAGGTGTCAACTACCATCCAGGCACCAACAGGTCTTATAATTGTCTTACGAGAGGC
70H1-5B1          GTGGAAGGTGTCAACTACCATCCAGGCACCAACAGGTCTTATAATTGTCTTACGAGAGGC
634H1-5B1         GTGGAAGGTGTCAACTACCATCCAGGCACCAACAGGTCTTATAATTGTCTTACGAGAGGC
                  ************************************************************
```

Figure 18. Alignment of multiple wheat genomic resources plus Sanger sequencing of flanking region of putative SNP located on the 5B1 sequence using genotypes displaying different alleles at the putative Kukri_c46570_214 SNP site. The numbers refer to wheat genotypes used in this study. Yellow color highlight letter represents the polymorphism between IWGSC sequence and some other genotypes. H1 refers to Hap-5B-RDMa-h1 and H2 refers to Hap-5B-RDMa-h2. The red highlight letters represent the allele of Kukri_c46570_214 SNP site.
