# Supplementary material for: Overcoming polyploidy pitfalls: a user guide for effective SNP conversion into KASP markers in wheat
Source: Theor Appl Genet. 2020 Jun 4;133(8):2413–30. doi: 10.1007/s00122-020-03608-x (PMC7360542; doi:10.1007/s00122-020-03608-x)
Supplement: Supplementary file 4 — 90k SNP chip discrimination plots for SNP probes located in haploblocks Hap-5B-RDMa and Hap-5B-RDMb for 213 genotypes of a wheat diversity panel (PDF 3639 kb) [file 122_2020_3608_MOESM4_ESM.pdf]

Supplementary Figure S1: SNP calls received from TraitGenetics, cluster plots produced from raw SNP chip data using the software GenomeStudio and comparison with publically available wheat sequence data and Sanger sequencing of regions flanking SNPs in up to six homeologuous and paraloguous copies of probe targets (5B1, 5B2, 5A1, 5A2, 5D1, 5D2). - indicates no copy detected (BLAST hits were filtered with an E-value threshold of 10e− 5).

**Hap-5B-RDMa**	GENE-2890_482 probe		Excalibur_c25522_755 probe		Kukri_c46570_214 probe	
Putative chromosome targets and homeologous/parlogous copies	SNPs called from alignment to public sequences	Similarity of 50 bp probe with IWGSC reference(+) or sequenced DNA(*)	SNPs called from alignment to public sequences	Similarity of 50 bp probe with IWGSC reference(+) or sequenced DNA(*)	SNPs called from alignment to public sequences	Similarity of 50 bp probe with IWGSC reference(+) or sequenced DNA(*)
5B - copy 1, Hap-5B-RDMa-h1	G	100%*	C	100%*	A	100%*
5B - copy 1, Hap-5B-RDMa-h2	A	98%*	T	98%*	G	98%*
5B - copy 2	-	-	C	90,19%+	G	100%+
5A- copy 1	A	96%*	T	96%+	G	98%+
5A - copy 2	-	-	C	96,07%+	-	-
5D - copy 1	A	100%*	C	86%+	G	100%+
5D -copy 2	-	-	C	92,15%+	-	-
SNP type	hemi		hemi		hemi	
Cluster positions from GenomeStudio using standard manifest file (putative nucleotide alleles for called cluster indicated)	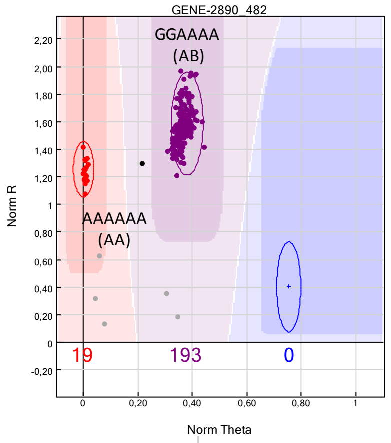		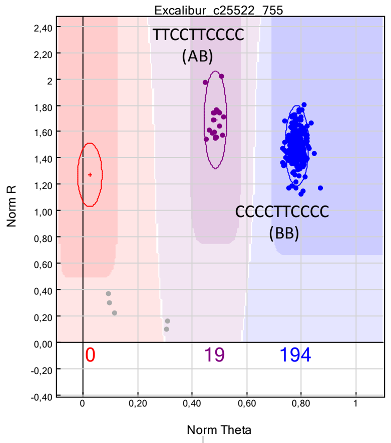		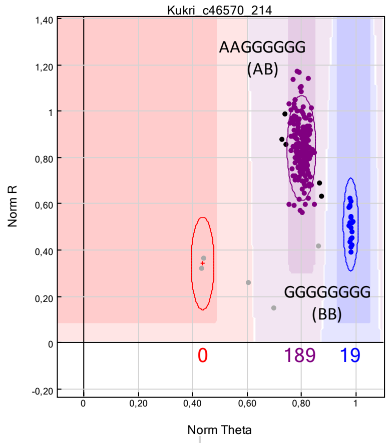	

**Hap-5B-RDMa**	RAC875_c12293_588 probe		RAC875_c18088_2222 probe		BobWhite_c43_86 probe	
Putative chromosome targets and homeologous/parlogous copies	SNPs called from alignment to public sequences	Similarity of 50 bp probe with IWGSC reference(+) or sequenced DNA(*)	SNPs called from alignment to public sequences	Similarity of 50 bp probe with IWGSC reference(+) or sequenced DNA(*)	SNPs called from alignment to public sequences	Similarity of 50 bp probe with IWGSC reference(+) or sequenced DNA(*)
5B - copy 1, Hap-5B-RDMa-h1	G	100%*	G	100%* and 98%*	G	100%* and 74%*
5B - copy 1, Hap-5B-RDMa-h2	A	98%*	A	98%*	A	98%*
5B - copy 2	-	-	-	-	-	-
5A- copy 1	G	96%*	G	98%+	A	86%+
5A - copy 2	-	-	-	-	-	-
5D - copy 1	A	98%*	G	98%+	A	56%+
5D -copy 2	-	-	-	-	-	-
SNP type	hemi		hemi		simple	
Cluster positions from GenomeStudio using standard manifest file (putative nucleotide alleles for called cluster indicated)	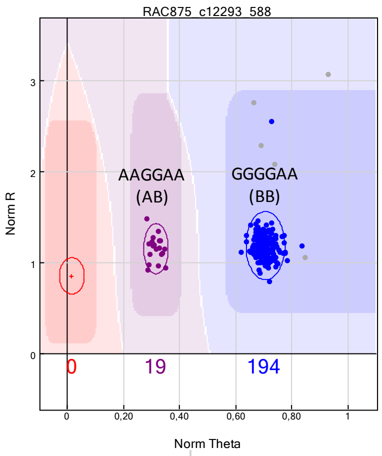		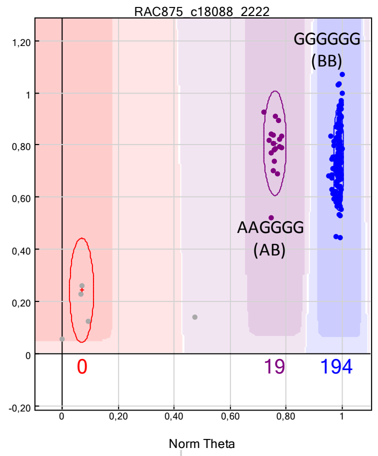		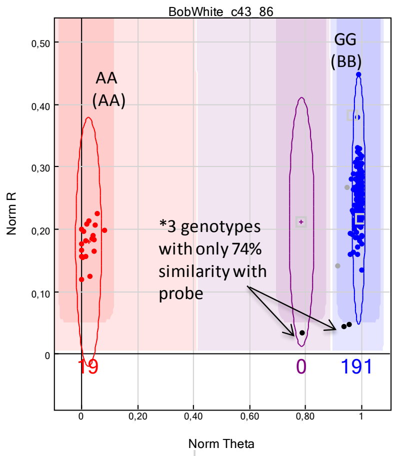	

<table>
<tr><th>Hap-5B-RDMa</th><th colspan="2">RAC875 c18088 950 probe</th><th colspan="2">RAC875 c24226 1356 probe</th><th colspan="2">Excalibur c60554 394 probe</th></tr>
<tr><td>Putative chromosome targets and homeologous/parlogous copies</td><td>SNPs called from alignment to public sequences</td><td>Similarity of 50 bp probe with IWGSC reference(+) or sequenced DNA(*)</td><td>SNPs called from alignment to public sequences</td><td>Similarity of 50 bp probe with IWGSC reference(+) or sequenced DNA(*)</td><td>SNPs called from alignment to public sequences</td><td>Similarity of 50 bp probe with IWGSC reference(+) or sequenced DNA(*)</td></tr>
<tr><td>5B - copy 1, Hap-5B-RDMa-h1</td><td>C</td><td>100%*</td><td>A</td><td>100%*</td><td>G</td><td>100%*</td></tr>
<tr><td>5B - copy 1, Hap-5B-RDMa-h2</td><td>T</td><td>100%*</td><td>C</td><td>100%*</td><td>A†</td><td>100%*</td></tr>
<tr><td>5B - copy 2</td><td>-</td><td>-</td><td>C</td><td>82%+</td><td>-</td><td>-</td></tr>
<tr><td>5A- copy 1</td><td>C</td><td>100%*</td><td>C</td><td>98%+</td><td>T</td><td>100%*</td></tr>
<tr><td>5A - copy 2</td><td>-</td><td>-</td><td>-</td><td>-</td><td>-</td><td>-</td></tr>
<tr><td>5D - copy 1</td><td>T</td><td>98%*</td><td>C</td><td>100%+</td><td>G</td><td>100%*</td></tr>
<tr><td>5D -copy 2</td><td>-</td><td>-</td><td>-</td><td>-</td><td>-</td><td>-</td></tr>
<tr><td>SNP type</td><td colspan="2">hemi</td><td colspan="2">hemi</td><td colspan="2">hemi</td></tr>
<tr><td>Cluster positions from GenomeStudio using standard manifest file (putative nucleotide alleles for called cluster indicated)</td><td colspan="2">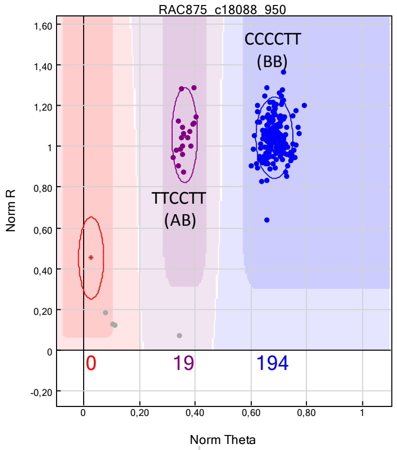
</td><td colspan="2">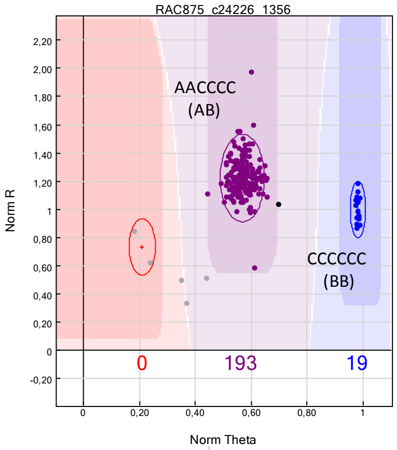
</td><td colspan="2">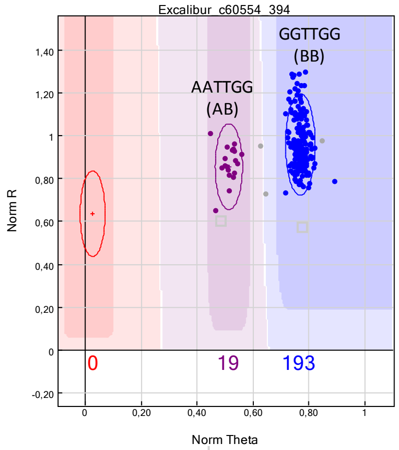
</td></tr>
</table>

† Sanger sequencing revealed for SNP Excalibur_c60554_394 a A/G polymorphism instead of reported T/G polymorphism called from 90K SNP array

**Hap-5B-RDMb**	BS00022231_51 probe		BS00022477_51 probe		BS00029852_51 probe	
Putative chromosome targets and homeologous/parlogous copies	SNPs called from alignment to public sequences	Similarity of 50 bp probe with IWGSC reference(+) or sequenced DNA(*)	SNPs called from alignment to public sequences	Similarity of 50 bp probe with IWGSC reference(+) or sequenced DNA(*)	SNPs called from alignment to public sequences	Similarity of 50 bp probe with IWGSC reference(+) or sequenced DNA(*)
5B - copy 1, Hap-5B-RDMb-h1	G	100%*	A	100%*	G	100%*
5B - copy 1, Hap-5B-RDMb-h2	A	100%*	G	100%*	T	100%*
5B - copy 1, Hap-5B-RDMb-h3	G	100%*	G	100%*	T	100%*
5B - copy 1, Hap-5B-RDMb-h8	G	100%*	A	100%*	G	100%*
5A - copy 1	-	-	G	98%*	T	46%+
5D - copy 1	-	-	G	96%*	T	79%+
SNP type	simple		hemi		simple	
Cluster positions from GenomeStudio using standard manifest file (putative nucleotide alleles for called cluster indicated)	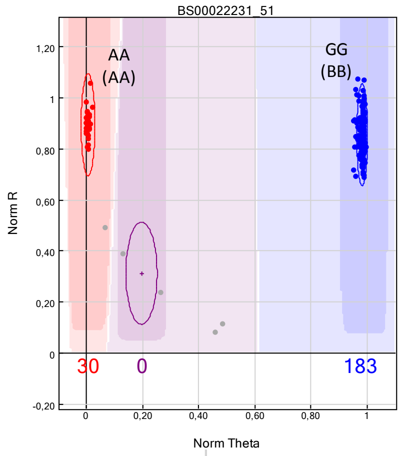		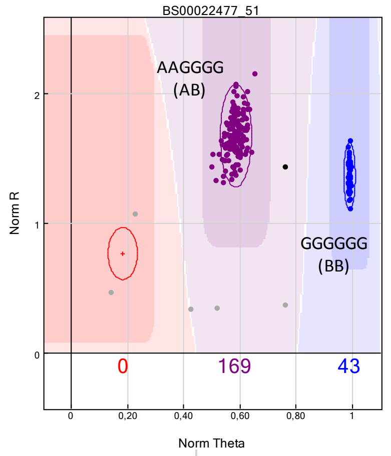		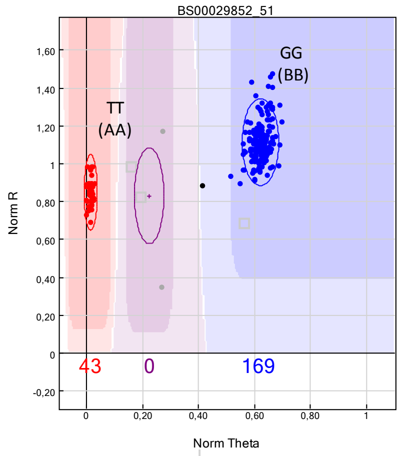	

**Hap-5B-RDMb**	BS00110293_51 probe		IACX6288 probe		Tdurum_contig48959_1172 probe	
Putative chromosome targets and homeologous/parlogous copies	SNPs called from alignment to public sequences	Similarity of 50 bp probe with IWGSC reference(+) or sequenced DNA(*)	SNPs called from alignment to public sequences	Similarity of 50 bp probe with IWGSC reference(+) or sequenced DNA(*)	SNPs called from alignment to public sequences	Similarity of 50 bp probe with IWGSC reference(+) or sequenced DNA(*)
5B - copy 1, Hap-5B-RDMb-h1	A	100%*	T	94,91%*	G	100%*
5B - copy 1, Hap-5B-RDMb-h2	G	100%*	C	96,6%*	G	100%*
5B - copy 1, Hap-5B-RDMb-h3	G	98%*	C	96,6%*	T	100%*
5B - copy 1, Hap-5B-RDMb-h8	G	98%*	C	97%*	T	100%*
5A - copy 1	-	-	C	100%+	-	-
5D - copy 1	-	-	C	98%+	-	-
SNP type	unclear (densely spaced homozygous subclusters)		hemi		simple	
Cluster positions from GenomeStudio using standard manifest file (putative nucleotide alleles for called cluster indicated)	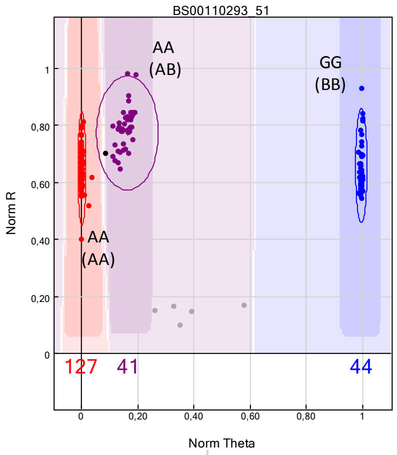		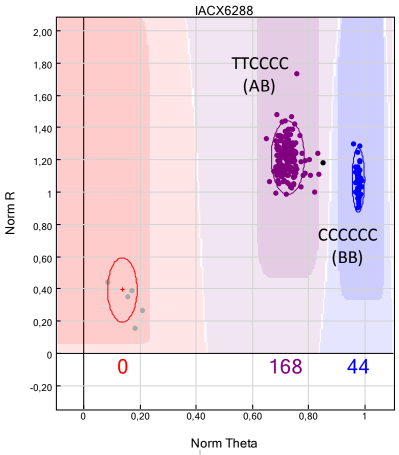		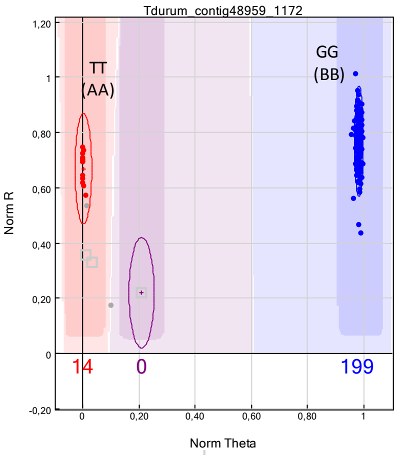	
