# Supplementary material for: Overcoming polyploidy pitfalls: a user guide for effective SNP conversion into KASP markers in wheat
Source: Theor Appl Genet. 2020 Jun 4;133(8):2413–30. doi: 10.1007/s00122-020-03608-x (PMC7360542; doi:10.1007/s00122-020-03608-x)
Supplement: Supplementary file 5 — KASP discrimination plots for KASP assays located in haploblocks Hap-5B-RDMa and Hap-5B-RDMb for 213 genotypes of a wheat diversity panel (PDF 193 kb) [file 122_2020_3608_MOESM5_ESM.pdf]

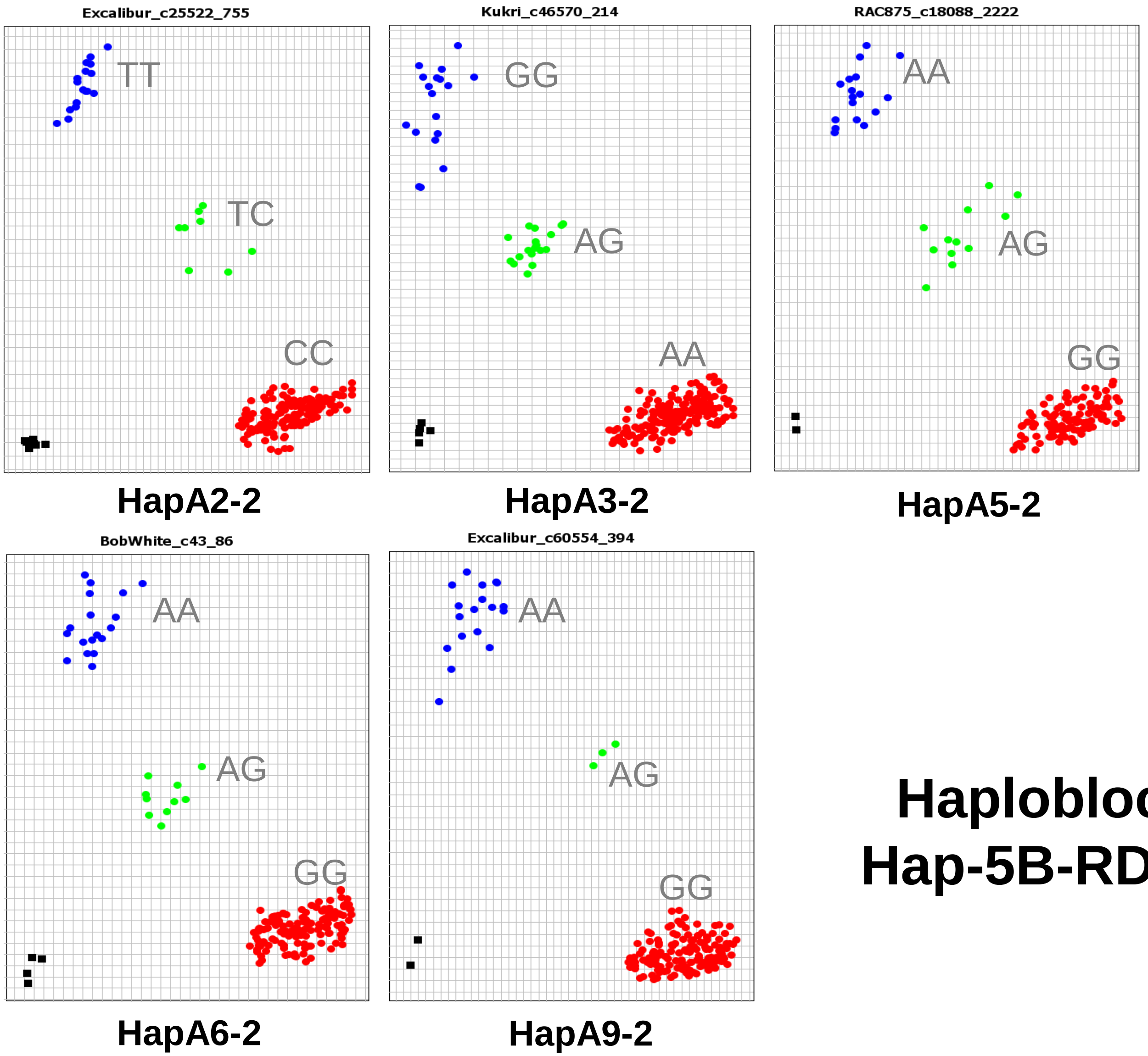

Haploblock
Hap-5B-RDMa

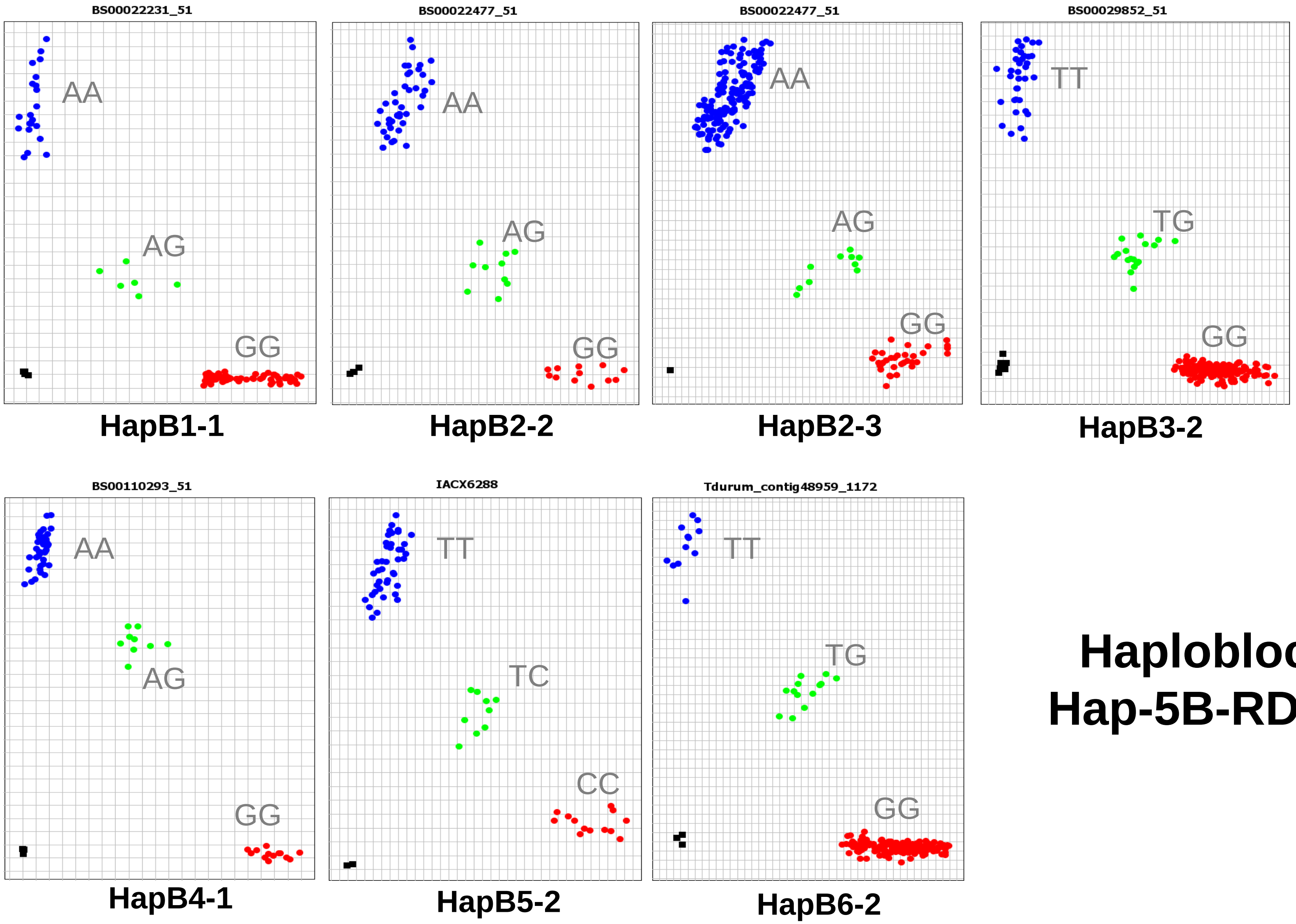

Haploblock Hap-5B-RDMb

Two thermo-cycling programmes were used with PACE-IR Genotyping Master Mix with a low ROX level (3cr bioscience, Harlow, UK) for genotyping of a diversity panel of 213 wheat accession using the developed KASP assays

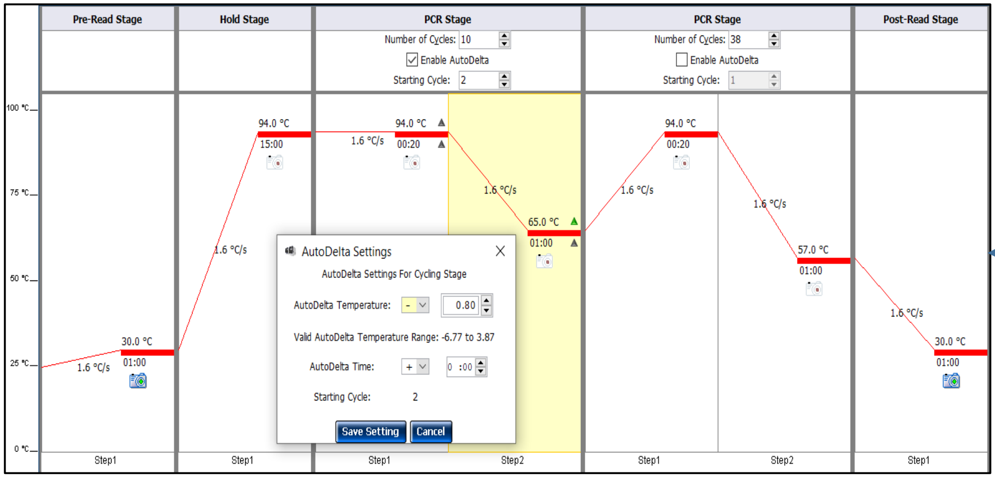

HapA3-2
HapB2-3

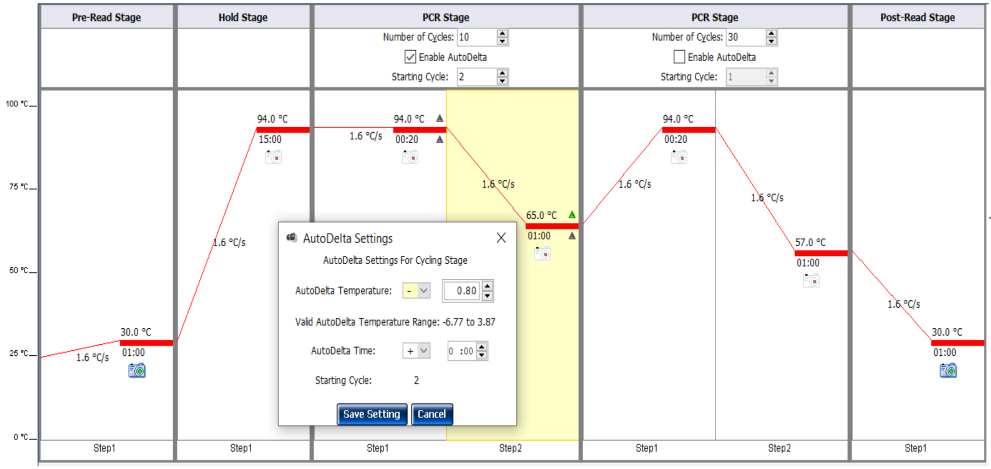

HapB1-1
HapA9-2 HapB2-2
HapA6-2 HapB3-2
HapA5-2 HapB4-1
HapA2-2 HapB5-2
HapB6-1
HapB6-2
